# Progressive endocannabinoid system dysregulation in autosomal dominant polycystic kidney disease

**DOI:** 10.1186/s10020-026-01457-w

**Published:** 2026-03-16

**Authors:** Shridhar Betkar, Alina Nemirovski, Shmuel Ruppo, Liad Hinden, Joseph Tam

**Affiliations:** 1 Obesity and Metabolism Laboratory, Institute for Drug Research, School of Pharmacy, Faculty of Medicine, The Hebrew University of Jerusalem, POB 12065, Jerusalem, 9112001 Israel; 2 Info-CORE, Bioinformatics Unit of the I-CORE, The Hebrew University of Jerusalem, Jerusalem, Israel

**Keywords:** Autosomal dominant polycystic kidney disease, Chronic kidney disease, Endocannabinoid system, Cannabinoid-1 receptor, Anandamide

## Abstract

**Background:**

Autosomal dominant polycystic kidney disease (ADPKD) is characterized by progressive cyst formation, inflammation, and metabolic dysregulation. The endocannabinoid system (ECS), particularly the cannabinoid-1 receptor (CB_1_R), regulates renal metabolism and inflammatory signaling, yet its role in ADPKD remains largely unexplored.

**Methods:**

We analyzed publicly available human kidney transcriptomic datasets (bulk microarray GSE7869; single-nucleus RNA-sequencing from ADPKD GSE185948, and diabetic kidney disease cohorts GSE195460) and validated findings in ADPKD patient kidney tissue versus non-cystic controls using quantitative PCR, liquid chromatography-tandem mass spectrometry, and Western blotting. Longitudinal disease progression was evaluated in *Pkd1*^RC/RC^ mice at 3, 6, 9, and 12 months, with comprehensive assessment of ECS components, endocannabinoid (eCB) levels, and kidney function parameters. Correlation examined associations between ECS markers and disease severity.

**Results:**

Human ADPKD kidneys demonstrated consistent upregulation of *CNR1* transcripts across platforms, with single-nucleus analysis revealing enrichment in proximal tubule-derived populations including failed-repair proximal tubule cells. ADPKD tissue exhibited significant reductions in key ECS-metabolizing enzymes (*FAAH*, *NAPEPLD*, *MGLL*) and marked depletion of eCB ligands anandamide (AEA) and 2-arachidonoylglycerol (2-AG). In contrast, diabetic kidney disease showed minimal ECS alterations, indicating ADPKD-specific dysregulation. *Pkd1*^RC/RC^ mice recapitulated human findings, with *Cnr1* upregulation beginning at 6 months and significant AEA/*N*-oleoylethanolamine (OEA) depletion at 9–12 months. CB_1_R protein elevation preceded ligand depletion, suggesting progressive receptor sensitization. Correlation analyses revealed robust associations between CB_1_R/enzyme expression, eCB depletion, and declining kidney function (kidney weight-to-body weight ratio, blood urea nitrogen, and creatinine clearance).

**Conclusions:**

ADPKD kidneys exhibit disease-specific dysregulation of the ECS, characterized by increased CB_1_R expression accompanied by paradoxical depletion of eCB ligands. These alterations correlate with cyst burden and functional decline across human and murine disease stages, identifying the ECS as a prominently affected pathway during ADPKD progression. While our findings establish a strong association between ECS dysregulation and disease severity, whether altered CB_1_R signaling represents a causal driver of cystogenesis or a secondary, yet therapeutically targetable component of the cystic and injury response will require direct genetic or pharmacologic modulation of CB_1_R/ECS signaling.

**Supplementary Information:**

The online version contains supplementary material available at 10.1186/s10020-026-01457-w.

## Translational statement

This study establishes progressive endocannabinoid system dysregulation as a novel pathogenic feature of ADPKD, with CB_1_R upregulation occurring in metabolically stressed tubular epithelium. These findings provide mechanistic rationale for targeting CB_1_R signaling in ADPKD and suggest potential synergy with emerging metabolic interventions.

## Introduction

Autosomal dominant polycystic kidney disease (ADPKD) is among the most prevalent genetic disorders, affecting approximately 12 million people globally, or about 1 in 2,000 individuals (A.W.G. KDIGO 2025). The disease is characterized by progressive development of fluid-filled cysts throughout the kidneys, leading to organ enlargement, dysfunction, and eventual end-stage kidney disease (ESKD), typically by the fifth or sixth decade of life. ADPKD is caused by mutations in either the *PKD1* (encoding polycystin-1 [PC1]; ~ 85% of cases) or *PKD2* genes (encoding polycystin-2 [PC2]; ~ 15% of cases) (Kim and Park 2016; Nobakht et al. 2020). Although PC1 and PC2 are believed to function as ciliary mechanosensors regulating calcium and fluid homeostasis, the full mechanistic understanding of how their dysfunction drives cystogenesis remains incomplete (Bergmann et al. 2018). To date, Tolvaptan remains the only approved disease-modifying therapy for ADPKD (Muller et al. 2022). However, substantial therapeutic progress has been made in recent years, with multiple alternative strategies, including metabolic interventions as well as emerging gene/RNA- and nanomedicine-based approaches, advancing into clinical trials (Chen, et al. 2025; Maciejczyk and Niemczyk 2025; Uchiyama et al. 2025). Accumulating evidence indicates that PC1 expression is therapeutically modifiable: kidney-targeted anti-microRNA approaches and disruption of microRNA-mediated cis-inhibition of PKD1/PKD2 transcripts have been shown to increase PC1/PC2 expression and attenuate cyst growth in preclinical models (Lee et al. 2019; Lakhia et al. 2022; Lakhia et al. 2025; ClinicalTrials.gov. 2020, 2019). While these approaches are promising, they are still in early stages of development and have not yet translated into broadly available clinical therapies. Altogether, these developments highlight both the expanding therapeutic landscape and the persistent need to identify and validate additional strategies capable of effectively inhibiting ADPKD progression.

Dysregulated cellular metabolism is now recognized as a fundamental driver of ADPKD progression (Padovano et al. 2018; Nowak and Hopp 2020; Rowe et al. 2013; Riwanto et al. 2016; Hogan and Masyuk 2023). Multiple studies have documented that metabolic disorders accelerate early-stage disease (Nowak et al. 2018; Nowak et al. 2021), and impaired glucose metabolism has been consistently observed in both murine PKD models and human ADPKD kidneys (Rowe et al. 2013; Riwanto et al. 2016; Kraus et al. 2016; Sas et al. 2015). Pharmacological inhibition of glucose metabolism (Chiaravalli et al. 2016) and caloric restriction interentions (Warner et al. 2016; Kipp et al. 2016) have demonstrated benefits in slowing cyst growth preclinically; however, these approaches impose significant lifestyle burdens on ADPKD patients. This treatment gap underscores the need for novel therapeutic strategies targeting the metabolic aberrations that fuel cyst expansion.

The endocannabinoid system (ECS) is an endogenous signaling network that plays central roles in metabolic homeostasis and tissue remodeling. The ECS comprises endocannabinoid (eCB) ligands (primarily anandamide [AEA] and 2-arachidonoylglycerol [2-AG]), two primary G-protein-coupled receptors (cannabinoid-1 receptor [CB_1_R], and cannabinoid-2 receptor [CB_2_R]), and enzymes responsible for eCB synthesis and degradation. CB_1_R is predominantly expressed in the central nervous system but is also present in peripheral tissues including the kidney, where it modulates hemodynamics, tubular reabsorption, protein excretion, and immunomodulation (Tam et al. 2018; Moreno et al. 2021). Increasing evidence links ECS dysregulation to multiple renal pathologies including renal injury (Dao and François 2021; Mukhopadhyay et al. 2010; Tam 2016; Trojnar et al. 2020; Jourdan et al. 2014; Jourdan et al. 2018). Yet, its specific role in ADPKD has not been systematically investigated.

A compelling rationale links CB_1_R signaling to ADPKD-relevant metabolic pathways. Our laboratory has shown that proximal tubular CB_1_R activation regulates two critical energy sensors: AMP-activated protein kinase (AMPK) (Udi et al. 2017), which restrains cell proliferation through inhibition of mammalian target of rapamycin complex 1 (mTORC1), and mTORC1 itself (Hinden et al. 2022), which promotes anabolic growth and cysts expension (Song et al. 2020; Podrini et al. 2020; Caplan 2022; Schrier and Levi 2010). Elevated mTORC1 activity is causally linked to cystogenesis and is currently being targeted in clinical trials (Margaria et al. 2020). Conversely, AMPK activation suppresses mTORC1, and additionally modulates cAMP signaling and electrolyte transport, processes that directly involved in cyst fluid secretion (Caplan 2022; Reiterova and Tesar 2022). These convergent lines of evidence suggest that dysregulated CB_1_R signaling could centrally contribute to the metabolic reprogramming and epithelial dysfunction characteristic of ADPKD.

To date, only a single clinical study has assessed circulating eCB levels in ADPKD patients, reporting reductions in both 2-AG and AEA (Klawitter et al. 2022). However, circulating eCB concentrations may not reliably reflect kidney-specific eCB activity (‘tone’) owing to the dynamic nature of these lipid mediators and their synthesis across multiple tissues (Hillard 2018). Therefore, characterizing kidney-specific ECS alterations across the disease timeline is essential for understanding ADPKD pathogenesis and identifying novel intervention targets.

Here, we provide the first comprehensive multi-platform analysis of ECS behavior in ADPKD, integrating human bulk and single-nucleus transcriptomics, tissue biochemistry from ADPKD kidneys, and longitudinal profiling in a *Pkd1*^RC/RC^ mouse model. We hypothesized that ECS components are progressively dysregulated during ADPKD and that this dysregulation, particularly altered CB_1_R signaling, contributes to disease progression. Our findings establish ECS dysfunction as a previously unrecognized hallmark of ADPKD pathophysiology and identify CB_1_R/ECS as a promising target that warrants mechanistic testing in future studies.

## Methods

### Microarray data analysis—human ADPKD (GSE7869)

Publicly available microarray gene expression data from ADPKD kidney biopsies (GSE7869 (Rowe et al. 2013; Song et al. 2009)); Affymetrix Human Genome U133 Plus 2.0 platform) were obtained from the NCBI Gene Expression Omnibus (GEO). The dataset comprised non-cystic control cortex (*n* = 6), minimally cystic ADPKD tissue (PKDm, *n* = 5), and advanced cystic ADPKD tissue (PKD, *n* = 8). Differential gene expression analysis was performed using GEO2R with limma-based linear modeling and Benjamini–Hochberg false discovery rate (FDR) correction. ECS-related genes (*CNR1*, *CNR2*, *NAPEPLD*, *FAAH*, *DAGLA*, *DAGLB*, *MGLL*) were extracted for focused analysis.

### Single-nucleus RNA-sequencing analysis: human ADPKD and DKD

#### ADPKD Dataset

snRNA-seq data (GSE185948 (Muto et al. 2022)) from ADPKD patients (*n* = 8) and healthy controls (*n* = 5) were obtained from GEO and analyzed using the Humphreys Lab Kidney Interactive Transcriptomics (KIT) portal (https://humphreyslab.com/SingleCell/). Samples were processed individually using 10 × Genomics Chromium Single Cell 3′ v3 chemistry.

#### DKD Dataset

snRNA-seq data (GSE195460 (Wilson et al. 2022)) from diabetic nephropathy patients (*n* = 5) and controls (*n* = 6) were similarly obtained and processed.

#### Analysis pipeline

Filtered gene-barcode count matrices generated by Cell Ranger were downloaded from GEO and imported into R (v4.4) for analysis using Seurat (v5.0). Quality control excluded nuclei with < 200 detected genes or > 10% mitochondrial RNA, and genes detected in < 3 nuclei. Data were normalized and scaled using Seurat’s standard workflow, and samples were integrated using Seurat’s reciprocal PCA (RPCA), followed by unsupervised clustering (resolution = 0.5) and UMAP visualization. Expression of seven ECS genes (*CNR1*, *CNR2*, *DAGLA*, *DAGLB*, *FAAH*, *MGLL*, *NAPEPLD*) was quantified as Log₂-transformed average values per sample and cell type. Pseudo-bulk expression profiles were generated by averaging expression values across all cell types per sample. Group comparisons employed Mann–Whitney U tests (*p* < 0.05 significant). Dot plots and UMAP overlays were obtained from KIT portal; bar and dot plots were generated in GraphPad Prism (v10.4).

### Human ADPKD kidney tissue

Human ADPKD cyst tissue samples (*n* = 17) and non-diseased human kidney (NHK) controls (*n* = 5) were kindly obtained from Prof. Owen M. Woodward at the University of Maryland School of Medicine via the NIDDK-sponsored Polycystic Kidney Disease Research Resource Consortium (PKD-RRC). ADPKD samples consisted of cyst-derived tissue collected from male and female patients, while control samples were derived from histologically normal renal cortex or medulla. All samples were de-identified prior to transfer, and no protected health information was provided. Tissue procurement and distribution were conducted in accordance with institutional review board approvals at the source institution.

### Animals and experimental protocol

All procedures were approved by the Hebrew University Institutional Animal Care and Use Committee (AAALAC accreditation #1285; Ethics MD-22–16,757-4) and followed ARRIVE guidelines (Kilkenny et al. 2010). Male and female mice on a C57BL/6 J background were housed under specific pathogen‐free conditions, with standard chow (Teklad #2918) and water *ad libitum*.

To investigate ECS dynamics in ADPKD, we employed *Pkd1*^RC/RC^ mice harboring a polymorphic R3277C point mutation in the PKD1 gene (kindly provided from Prof. Peter C. Harris, Mayo Foundation for Medical Education and Research, USA). Littermates were segregated by sex at 3 weeks and monitored longitudinally at 3, 6, 9, and 12 months. One week prior to euthanasia, 23-h urine samples were collected using mouse metabolic cages (CCS2000 Chiller System, Hatteras Instruments, NC, USA). Body weights were recorded immediately before euthanasia by cervical dislocation under anesthesia. Kidneys were harvested, weighed, and either snap-frozen for biochemical analyses or fixed in 4% buffered formalin for histology. Blood was collected via retro-orbital sampling under anesthesia. All mouse terminal procedures and sample collections from WT (*n* = 5) and *Pkd1*^RC/RC^ (*n* = 5–12) per group per timepoint were performed during the light phase, between 08:00 and 12:00, with a maximum variation of 2 h between animals, in order to minimize circadian variation in eCB levels.

### Blood and urine biochemistry

Serum and urine samples from WT (*n* = 5) and *Pkd1*^RC/RC^ (*n* = 5–12) per group per timepoint were analyzed using a Cobas C-111 chemistry analyzer (Roche Diagnostics, Basel, Switzerland) for urea, glucose, and creatinine. Blood urea nitrogen (BUN) was calculated as: BUN (mg/dL) = Urea (mmol/L) × 2.801. Creatinine clearance (CCr) was calculated as: CCr (mL/h) = [Urine creatinine (mg/dL) × Urine volume (mL)]/[Serum creatinine (mg/dL0 × 23 h).

### Endocannabinoid extraction and LC–MS/MS quantification

eCBs from WT (*n* = 5) and *Pkd1*^RC/RC^ (*n* = 5–12) were extracted and quantified by stable isotope dilution liquid chromatography/tandem mass spectrometry (LC–MS/MS) as previously described with minor modifications (Hinden et al. 2022). Kidney cortex tissues were homogenized in ice-cold Tris Buffer, protein concentration was determined, and samples underwent chloroform/methanol extraction (three sequential extractions). Organic phases were pooled, dried, reconstituted, and analyzed on a Sciex QTRAP® 6500^+^ mass spectrometer coupled to a Shimadzu UHPLC System. Chromatographic separation used a Kinetex 2.6 µm C18 column (100 × 2.1, Phenomenex) with gradient elution (0.1% formic acid in water and 0.1% formic acid in acetonitrile). eCBs were detected in positive ion mode using electron spray ionization (ESI) and multiple reaction monitoring (MRM), with d_4_-AEA and d_5_−2-AG as internal standards. Concentrations of *N*-arachidonoylethanolamine (AEA), 2-arachidonoylglycerol (2-AG), *N*-oleoylethanolamine (OEA), *N*-palmitoylethanolamine (PEA), and arachidonic acid (AA) were determined against standard curves and normalized to protein concentration. MRM transitions and mass spectrometry parameters are provided in Supplementary Table 1.

### Real-time quantitative PCR

Total kidney RNA from WT (*n* = 5) and *Pkd1*^RC/RC^ (*n* = 5–12) was extracted using Bio-Tri RNA lysis buffer (Bio-Lab), treated with DNase I (Thermo Scientific, IL, USA), and reverse-transcribed using the iScript cDNA synthesis kit (Bio-Rad). Real-time PCR was performed using iTaq Universal SYBR Green Supermix (Bio-Rad) on a CFX connect system (Bio-Rad). Gene expression was normalized to HPRT1 (human samples) or ubiquitin C (*Ubc*; mouse samples). Primer sequences are provided in Supplementary Tables 2 and 3.

### Immunohistochemistry

Kidney tissues from WT (*n* = 3) and *Pkd1*^RC/RC^ (*n* = 3–5) were fixed in buffered 4% formalin for 48 h and then embedded in paraffin. Sections were deparaffinized and hydrated. Heat-mediated antigen retrieval was performed with 10 mM citrate buffer pH 6.0 (Thermo Scientific, IL, USA). Endogenous peroxide was inhibited by incubating with a freshly prepared 3% H_2_O_2_ solution in MeOH. Unspecific antigens were blocked by incubating sections for 1 h with 2.5% horse serum (VE-S-2000, Vector Laboratories). Sections were stained with rabbit anti-CB_1_R antibody (1:200, 10006590, Cayman) followed by a goat anti-rabbit HRP conjugate (ImmPRESS™, Vector laboratories). Color was developed after an incubation with 3,3'-Diaminobenzidine (DAB) substrate [ImmPACT DAB Peroxidase (HRP) Substrate, SK-4105, Vector Laboratories], followed by hematoxylin counterstaining and mounting (Vecmount H-5000, Vector laboratories). Images were captured using an AxioCam ICc5 color camera mounted on an Axio Scope.A1 light microscope (Zeiss).

### Fluorescence immunohistochemistry

Kidney sections from WT (*n* = 3) and *Pkd1*^RC/RC^ (*n* = 3–5) per group per timepoint were deparaffinized, rehydrated, and subjected to heat-mediated antigen retrieval in 10 mM citrate buffer (pH 6.0; Thermo Scientific, IL, USA). Non-specific binding was blocked with 5% goat serum (Vector Laboratories) for 1 h. Sections were incubated with rabbit anti-CB_1_R (1:500, 301214, Immunogenes) followed by goat anti-rabbit-AlexaFluor488 (1:500, ab150077, Abcam). Sections were mounted with DAPI-containing medium (Vector Laboratories) and imaged using an LSM 700 confocal imaging system (Zeiss). Relative fluorescence intensity (RFI) was quantified using ImageJ (NIH).

### Protein extraction and western blotting analysis

Kidney tissue samples from WT (*n* = 5) and *Pkd1*^RC/RC^ (*n* = 5–12) were homogenized in ice-cold RIPA lysis buffer (25 mM Tris–HCl pH 7.6, 150 mM NaCl, 1% NP-40, 1% sodium deoxycholate, 0.1% SDS) supplemented with protease and phosphatase inhibitor cocktails using the BulletBlender® with zirconium oxide beads (Next Advanced, Inc., NY, USA). Lysates were cleared by centrifugation at 12,000 × g for 10 min at 4 °C, and protein concentrations were determined using a bicinchoninic acid (BCA) assay with the Pierce™ BCA Protein Assay Kit (Thermo Scientific, IL, USA). Equal amounts of protein were mixed with Laemmli sample buffer, denatured at 95 °C for 5 min, and separated by SDS-PAGE on 8–18% polyacrylamide gels. Proteins were transferred onto nitrocellulose membranes using the Trans-Blot® Turbo™ Transfer System (Bio-Rad, CA). Membranes were stained with Ponceau to verify equal protein loading and transfer efficiency, followed by blocking in 5% non-fat milk or 5% bovine serum albumin (BSA) in Tris-buffered saline containing 0.1% Tween-20 (TBS-T) for 1 h at room temperature. Membranes were incubated overnight at 4 °C with primary antibodies (Supplementary Table 4) diluted in blocking buffer. After washing with TBS-T, membranes were incubated with appropriate HRP-conjugated secondary antibodies for 1 h at room temperature. Chemiluminescence detection was performed using Clarity™ Western ECL Blotting Substrate (Bio-Rad, CA) and imaged with ChemiDoc™ Touch Imaging System (Bio-Rad, CA). Band intensities were quantified using Image lab software and normalized to total protein levels as assessed by Ponceau staining (or to housekeeping proteins where indicated). Data is presented as relative protein abundance compared with control samples.

### Histology

Kidney tissues from WT (*n* = 4) and *Pkd1*^RC/RC^ (*n* = 5) were fixed in 10% neutral buffered formalin, processed, and embedded in paraffin according to standard histological procedures. Paraffin-embedded tissue blocks were sectioned at 4–5 μm thickness using a rotary microtome and mounted on glass slides. Sections were deparaffinized in xylene and rehydrated through a graded ethanol series to distilled water. Sections were stained with hematoxylin and eosin (H&E), dehydrated through graded ethanol, cleared in xylene, and cover slipped using a permanent mounting medium. Stained sections were examined using a bright-field light microscope, and representative images were acquired under identical imaging conditions for all experimental groups. Histological quantification was performed using QuPath software. Cyst burden was assessed by calculating the cystic index (CI), defined as: [CI (%) = 100 × (mean cyst area × cyst count)/total tissue area], where mean cyst area represents the average area of individual cysts and total tissue area represents the average area of the analyzed renal tissue section.

### Statistical analysis

Data are expressed as mean ± SEM. Statistical analyses were performed using GraphPad Prism (v10.4). For multi-group comparisons involving genotype, sex, or time, two-way ANOVA followed by Sidak’s post-hoc multiple comparisons test was performed. Two-group comparisons employed unpaired *t*-test or Mann–Whitney U tests (human samples). Correlations were assessed using Spearman’s rank correlation. Statistical significance was defined as *p* < 0.05. For snRNA-seq data, FDR-adjusted *p* < 0.05 was considered significant.

## Results

### Multi-platform transcriptomic evidence of ECS dysregulation in human ADPKD

A previous clinical study suggested dysregulation of circulating eCBs in ADPKD patients (Klawitter et al. 2022). However, circulating eCB levels may not reliably reflect kidney-specific ECS activity or ‘tone’, given their dynamic nature and diverse tissue sources (Hillard 2018). To accurately characterize kidney-specific ECS alterations in ADPKD, we first analyzed microarray-based transcriptomic data from the GSE7869 dataset, comprising healthy kidney cortex samples, minimally cystic ADPKD tissue (PKDm), and advanced cystic ADPKD tissue (PKD). Sample-level expression visualization (Supplementary Figure S1) demonstrated high intra-group consistency and progressive dysregulation across disease severity stages.

**Fig. 1:**
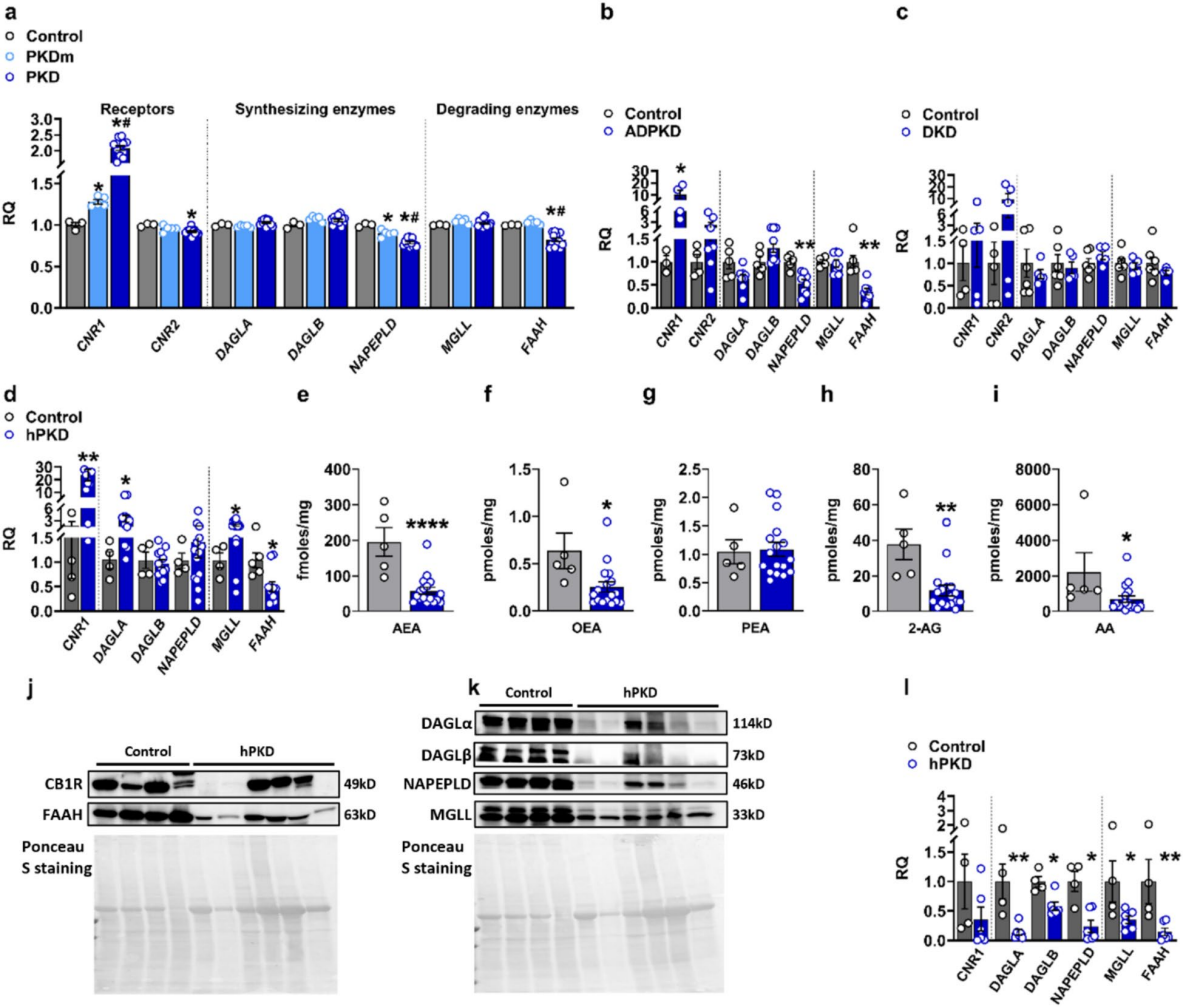
ECS components are progressively dysregulated in human ADPKD kidney tissue. **a** Microarray analysis (GSE7869) of human kidney tissue shows stepwise increases in *CNR1* transcript from healthy cortex to minimally cystic (PKDm) and fully cystic (PKD) ADPKD tissue, with corresponding reductions in AEA-metabolizing enzymes *NAPEPLD* and *FAAH*. **b** Single-nucleus RNA-sequencing (snRNA-seq) analysis of human ADPKD kidneys (*n* = 8) versus healthy controls (*n* = 5) demonstrate consistent *CNR1* upregulation and marked downregulation of *NAPEPLD* and *FAAH*, while 2-AG-metabolizing enzymes remain largely unchanged. **c** snRNA-seq analysis of diabetic kidney disease (DKD; *n* = 5 patients; controls *n* = 6) reveals minimal alterations in *CNR1* and ECS-metabolizing enzymes. **d** Gene expression analysis by qPCR confirms *CNR1* upregulation in human ADPKD kidney tissue (*n* = 17) versus non-cystic nephrectomy controls (*n* = 5), with concurrent changes in ECS enzyme transcription. **e-i** eCB quantification by liquid chromatography-tandem mass spectrometry (LC–MS/MS) reveals significant depletion of tissue anandamide (AEA); **e**, *N*-oleoylethanolamine (OEA); **f**, 2-arachidonoylglycerol (2-AG); **h**, and arachidonic acid (AA); **i**, while *N*-palmitoylethanolamine (PEA); **g** remains unchanged. **j-l** Western blot analysis shows substantial inter-individual variability in CB_1_R protein levels without significant difference between ADPKD (*n* = 6) and control kidneys (*n* = 4), but significant reductions in DAGLα/β, NAPEPLD, MGLL and FAAH protein. Western blots were normalized to total proteins. Data represent mean ± SEM. Statistics of control versus PKD represented by * and control versus PKDm by #. Statistical significance assessed by Mann–Whitney U test or unpaired *t*-test: *p < 0.05, **p < 0.01, ***p < 0.001, ****p < 0.0001

Across the disease spectrum, we observed marked and progressive dysregulation of ECS-related genes. Notably, *CNR1* (encoding CB_1_R) expression was significantly elevated in PKDm samples compared with healthy controls, with further increases in late-stage cystic tissue, demonstrating stage-dependent amplification of CB_1_R signaling (Fig. 1a). In contrast, *CNR2* (encoding CB_2_R) showed only modest decrease in bulk microarray data, a result that is likely influenced by the cyst dissection protocol, which under-represents inflamed, immune-cell-rich regions where *CNR2* is highest, as suggested by its leukocyte-restricted pattern in snRNA-seq. Among ECS enzymes, only AEA biosynthesizing and degrading enzymes (*NAPEPLD* and *FAAH*) were significantly reduced in PKD (Fig. 1a). These changes collectively suggested dysregulated ECS in ADPKD progression.

To examine ECS alterations with cellular precision, we interrogated single-nucleus RNA-sequencing data (snRNA-seq) from ADPKD (*n* = 8) and healthy control (*n* = 5) human renal cortex samples. Pseudo-bulk analysis revealed findings consistent with the GSE7869 dataset: *CNR1* expression was significantly elevated, while AEA-modulating enzymes *NAPEPLD* and *FAAH* exhibited marked reduction in ADPKD patients (Fig. 1b). Conversely, 2-AG-modulating enzymes (*DAGLA*, *DAGLB*, *MGLL*) showed minimal changes (Fig. 1b).

Cyst-lining epithelial cells were not resolved as a discrete transcriptional cluster in these datasets, and epithelial populations were defined based on nephron segment identity and injury-associated transcriptional states. Critically, cell-type resolved analysis revealed that ECS dysregulation is not uniformly distributed across the nephron but is enriched in the tubular compartment. Specifically, AEA-modulating enzymes displayed marked reductions across most cell types, while 2-AG-modulating enzymes showed altered expression in specific clusters (Supplementary Figure S2). Most notably, ECS dysregulation was predominantly enriched in proximal tubule-derived populations, particularly within the failed-repair proximal tubular cell (FR-PTC) population (Supplementary Figure S2). FR-PTCs represent a maladaptive epithelial state characterized by metabolic stress, dedifferentiation, and proliferative capacity; processes closely associated with cystic remodeling. Because a distinct “cyst epithelium” cluster is not separable in these datasets, our findings should be interpreted as ECS dysregulation in maladaptive tubular states enriched in ADPKD rather than as uniquely cyst-epithelium-specific.

### ECS dysregulation is ADPKD-specific, not a generic CKD feature

To determine whether ECS dysregulation represents a general response to chronic kidney disease (CKD) or is ADPKD-specific, we analyzed snRNA-seq data from diabetic kidney disease (DKD) patients using the identical analytical pipeline. In striking contrast to ADPKD, bulk gene expression analysis revealed non-significant changes in *CNR1* and *CNR2*, with minimal alterations in ECS-metabolizing enzymes in DKD patients (Fig. 1c). Cell-type resolved analysis confirmed absence of coordinated ECS dysregulation across DKD kidney compartments (Supplementary Figure S3). This disease comparison demonstrates that ECS disruption is not a universal feature of renal injury but appears specific to ADPKD pathology, strengthening the mechanistic relevance of our findings.

### Human tissue validation of ECS dysregulation

To validate transcriptomic findings at the tissue level and confirm ECS dysregulation in human ADPKD kidneys, we quantified ECS components in cortical kidney tissue from ADPKD patients (hPKD, *n* = 17) and non-cystic nephrectomy controls (NHK, *n* = 5). Assessment included transcriptional and protein expression of ECS receptors and enzymes, and eCB quantification. At the transcript level, *CNR1* transcription was significantly increased in ADPKD tissue compared with controls (Fig. 1d), consistent with human bulk microarray and single-nucleus transcriptomics. Among AEA-related enzymes, *NAPEPLD* did not change significantly, while *FAAH* was significantly decreased (Fig. 1d). For 2-AG-related enzymes, both *DAGLA* and *MGLL* showed significant increase, whereas *DAGLB* remained unchanged (Fig. 1d).

Next, tissue concentrations of eCBs were measured by LC–MS/MS. *N*-ethanol-amines, AEA and OEA were significantly depleted in ADPKD tissue (Fig. 1e, f), while PEA remained unchanged (Fig. 1g). 2-AG levels were significantly reduced in ADPKD kidneys relative to NHK controls (Fig. 1h), as was the eCB breakdown product AA (Fig. 1i). Notably, despite reduced *FAAH* expression, which would be expected to increase AEA and OEA levels, ADPKD kidneys exhibited global depletion of canonical eCBs and selected *N*-acylethanolamines. This paradox suggests that biosynthetic impairment or non-enzymatic consumption predominates over reduced degradation in regulating eCB levels during ADPKD.

To confirm this dysregulation at the protein level, Western blot analysis examined protein abundance of ECS components. In contrast to robust *CNR1* transcript upregulation, CB_1_R protein levels showed substantial inter-individual variability and did not differ significantly between ADPKD and control kidneys (Fig. 1j, l). However, NAPEPLD and FAAH, as well as DAGLA, DAGLB and MGLL protein levels were significantly reduced (Fig. 1j-i). Notably, the protein data for metabolic enzymes to some extent mirror, and in some cases accentuate, the dysregulation inferred from transcriptomics, providing orthogonal validation.

Taken together, across multiple analytical platforms, human ADPKD kidneys display a complex but coherent pattern of ECS disruption, characterized by: (i) increased *CNR1* transcription with paradoxically stable/variable CB_1_R protein; (ii) marked downregulation of key eCB-metabolizing enzymes; and (iii) selective depletion of AEA and 2-AG with marked reduction of OEA. This multi-platform convergence provides direct experimental evidence that ECS homeostasis is fundamentally altered in ADPKD kidney tissue.

### Establishment and pathological characterization of the longitudinal *Pkd1*^RC/RC^ mouse model

**Fig. 2:**
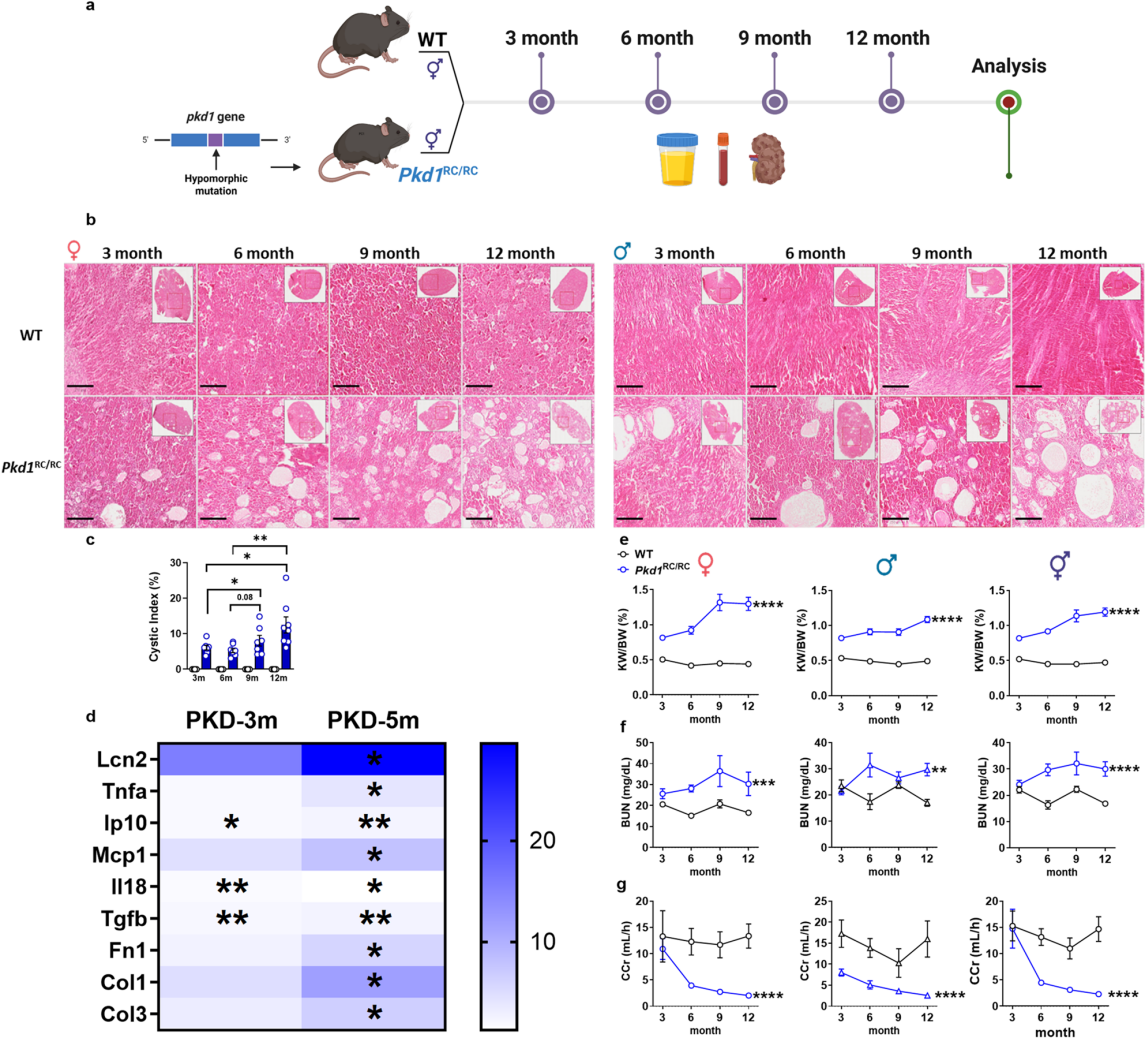
Establishment and pathological characterization of *Pkd1*^RC/RC^ Mice. **a** Experimental timeline showing longitudinal sampling at 3, 6, 9, and 12 months in wild-type (WT) and *Pkd1*^RC/RC^ mice, with collection of urine, blood, and kidney samples at each timepoint for biochemical, molecular, and histological analysis. **b** Representative hematoxylin and eosin (H&E)-stained kidney sections (× 20 magnification) from females (upper panels) and males (lower panels) showing progressive cyst development and tubular dilation in *Pkd1*^RC/RC^ mice compared with normal renal architecture in age-matched WT controls. Low-magnification overviews shown in insets. **c** Quantitative analysis of progressive cyst burden across the disease timeline, demonstrating significant age-dependent increase in both males and females (combined data shown). **d** Heatmap of renal gene expression for injury markers (*Lcn2*), pro-inflammatory cytokines (*Tnfa, Ip10, Mcp1, Il18b*), and pro-fibrotic mediators (*Tgfb, Fn1, Col1, Col3*) showing progressive activation of inflammatory and fibrotic signaling in *Pkd1*^RC/RC^ mice. **e–g** Longitudinal assessment of kidney function demonstrates significant age-dependent increases in kidney-to-body weight ratio (KW/BW; **e** and blood urea nitrogen (BUN; **f**, with parallel decline in creatinine clearance (CCr; **g** in female and male *Pkd1*^RC/RC^ mice versus WT controls. Data represent mean ± SEM from WT (*n* = 3–5) and *Pkd1*^RC/RC^ (*n* = 3–12) per group per timepoint. Statistical analysis: two-way ANOVA with Šidák post-hoc test for multiple comparisons. Statistical significance: *p < 0.05, **p < 0.01, ***p < 0.001, ****p < 0.0001 versus WT

Although human multi-omics datasets and tissue analyses provide compelling evidence of ECS dysregulation in ADPKD, they inherently capture disease at fixed, often advanced stages (ESKD). To investigate dynamic changes in ECS across disease progression, we employed *Pkd1*^RC/RC^ mice harboring a polymorphic R3277C point mutation in the PKD1 gene. This model recapitulates gradual-onset ADPKD and permits controlled longitudinal analysis. Wild-type (WT) and *Pkd1*^RC/RC^ mice were studied at 3, 6, 9, and 12 months of age. Blood, urine, and kidney samples were collected at each time point for biochemical, molecular, and eCB quantification analyses (Fig. 2a).

Longitudinal H&E staining revealed progressive cyst accumulation in *Pkd1*^RC/RC^ kidneys with age (Fig. 2b, c). Renal gene expression profiling demonstrated progressive activation of injury (*Lcn2*), inflammatory (*Tnfa*, *Ip10*, *Mcp1*, *Il1b*), and fibrotic (*Tgfb1*, *Fn1*, *Col1*, *Col3*) markers in *Pkd1*^RC/RC^ mice relative to WT (Fig. 2d). Longitudinal assessment of renal functional revealed significant age-dependent increase in kidney-to-body weight ratio (KW/BW; Fig. 2e) and BUN (Fig. 2f) with parallel decline in CCr (Fig. 2g) in both sexes. Together, these observations confirm that *Pkd1*^RC/RC^ mice undergo stepwise functional decline consistent with human ADPKD progression, validating this model for mechanistic investigation.

### Temporal ECS dysregulation parallels ADPKD progression in *Pkd1*^RC/RC^ mice

Longitudinal analysis of ECS-related genes and eCB levels in *Pkd1*^RC/RC^ mice revealed a stage-dependent pattern of dysregulation (Table 1). Consistent with human transcriptional data, *Cnr1* expression was significantly elevated in *Pkd1*^RC/RC^ mice beginning at 6 months of age and persisting through 12 months in both males and females. *Cnr2* expression was significantly downregulated in male *Pkd1*^RC/RC^ mice at 6 months and elevated in female *Pkd1*^RC/RC^ mice at 9 and 12 months, suggesting sex- and time-specific regulation of CB_2_R.

**Table 1:** Mouse kidney ECS-related gene expression and eCB levels

Males	3		6		9		12	
	WT	PKD	WT	PKD	WT	PKD	WT	PKD
*Cnr1*	1.03 ± 0.15	1.12 ± 0.29	1.04 ± 0.15	*3.43* ± 0.74*	1.07 ± 0.21	*6.11* ± 0.85***	1.07 ± 0.19	*4.93* ± 1.43*
*Cnr2*	1.02 ± 0.10	1.14 ± 0.28	1.02 ± 0.09	*0.70* ± 0.09*	1.01 ± 0.09	1.27 ± 0.14	1.03 ± 0.14	1.14 ± 0.21
*Dagla*	1.01 ± 0.08	1.47 ± 0.40	1.02 ± 0.11	1.22 ± 0.22	1.02 ± 0.10	*1.46* ± 0.12*	1.03 ± 0.11	1.04 ± 0.16
*Daglb*	1.10 ± 0.20	*2.13* ± 0.31*	1.00 ± 0.03	1.38 ± 0.25	1.16 ± 0.32	*2.56* ± 0.41*	1.04 ± 0.14	1.03 ± 0.19
*Napepld*	1.01 ± 0.08	1.21 ± 0.17	1.09 ± 0.23	1.15 ± 0.18	1.02 ± 0.09	*1.99* ± 0.27*	1.04 ± 0.15	1.11 ± 0.12
*Mgll*	1.09 ± 0.22	0.86 ± 0.17	1.01 ± 0.09	1.06 ± 0.11	1.02 ± 0.12	*1.56* ± 0.09**	1.04 ± 0.15	1.40 ± 0.28
*Faah*	1.02 ± 0.10	1.60 ± 0.26	1.01 ± 0.08	0.87 ± 0.18	1.10 ± 0.24	1.81 ± 0.25	1.03 ± 0.11	0.79 ± 0.10
2-AG	5.68 ± 2.78	7.87 ± 1.53	6.16 ± 1.98	8.69 ± 2.39	4.84 ± 1.21	8.59 ± 1.73	8.23 ± 2.41	9.85 ± 2.03
AA	235.22 ± 12.33	218.97 ± 18.94	219.78 ± 16.49	205.48 ± 15.55	277.59 ± 10.29	*199.60* ± 7.06****	225.51 ± 13.53	257.57 ± 32.45
AEA	185.23 ± 26.34	139.25 ± 20.19	124.35 ± 14.35	117.50 ± 9.80	255.43 ± 28.84	*117.70* ± 5.54***	168.79 ± 25.79	130.63 ± 29.97
OEA	321.09 ± 64.42	354.19 ± 98.51	322.96 ± 50.20	214.45 ± 31.70	478.12 ± 31.75	*191.05* ± 6.54****	363.37 ± 70.88	247.74 ± 55.49
PEA	2054.35 ± 372.54	1987.18 ± 547.28	1390.97 ± 224.95	1515.56 ± 257.80	1983.68 ± 211.04	*1310.31* ± 172.01*	1289.94 ± 280.62	1657.88 ± 219.18

Female	3		6		9		12	
	WT	PKD	WT	PKD	WT	PKD	WT	PKD
*Cnr1*	1.05 ± 0.14	2.00 ± 0.45	1.08 ± 0.22	*2.23* ± 0.31*	1.01 ± 0.07	*5.52* ± 1.33*	1.11 ± 0.26	*7.70* ± 2.12*
*Cnr2*	1.07 ± 0.20	0.68 ± 0.16	1.03 ± 0.12	1.24 ± 0.19	1.03 ± 0.12	*1.73* ± 0.17*	1.02 ± 0.09	*1.73* ± 0.14**
*Dagla*	1.06 ± 0.16	0.90 ± 0.19	1.06 ± 0.17	1.30 ± 0.16	1.00 ± 0.04	*1.99* ± 0.22**	1.02 ± 0.10	*1.60* ± 0.15*
*Daglb*	1.06 ± 0.19	0.94 ± 0.14	1.04 ± 0.14	*2.49* ± 0.21***	1.03 ± 0.12	*4.36* ± 0.38****	1.03 ± 0.13	*2.94* ± 0.13****
*Napepld*	1.02 ± 0.09	0.73 ± 0.13	1.02 ± 0.12	1.28 ± 0.14	1.04 ± 0.16	*1.54* ± 0.11*	1.03 ± 0.12	*1.70* ± 0.22*
*Mgll*	1.06 ± 0.20	1.06 ± 0.20	1.09 ± 0.22	*2.20* ± 0.32*	1.00 ± 0.04	*2.25* ± 0.40*	1.09 ± 0.22	*1.79* ± 0.16*
*Faah*	1.10 ± 0.26	1.31 ± 0.21	1.09 ± 0.22	*3.48* ± 0.42**	1.03 ± 0.12	*2.03* ± 0.36*	1.04 ± 0.14	1.54 ± 0.35
2-AG	4.61 ± 0.90	5.72 ± 1.25	5.07 ± 0.62	7.74 ± 1.24	4.96 ± 1.15	9.38 ± 1.74	5.69 ± 1.38	8.50 ± 1.30
AA	239.61 ± 10.70	228.72 ± 7.92	307.35 ± 38.16	251.73 ± 12.71	305.67 ± 38.57	242.38 ± 15.40	272.06 ± 12.19	238.82 ± 14.83
AEA	142.30 ± 16.72	128.13 ± 12.23	254.22 ± 65.00	139.06 ± 20.85	280.64 ± 64.00	*91.92* ± 23.64*	161.36 ± 12.14	*70.31* ± 10.16**
OEA	247.39 ± 21.20	291.14 ± 55.71	508.81 ± 121.50	279.41 ± 34.36	504.93 ± 118.78	*183.07* ± 19.37*	356.18 ± 25.81	*183.51* ± 13.99****
PEA	1836.45 ± 260.07	1606.55 ± 371.95	2253.46 ± 544.05	1960.54 ± 319.83	1562.19 ± 345.96	1590.13 ± 222.25	1424.88 ± 139.92	1730.32 ± 382.69

Units for ECS gene expression are relative quantification (RQ). Values are presented separately for wild-type (WT, *n* = 5) and *Pkd1*^RC/RC^ (*n* = 7) mice at each time point and for each sex, allowing direct comparison of eCB ligand levels (2-AG, AEA, OEA, PEA, AA) and ECS gene expression between genotypes. Units for eCB levels: 2-AG, AA, PEA (pmoles/mg), AEA, OEA (fmoles/mg). Student’s *t*-test- *P < 0.05, **P < 0.005, ***P < 0.001, ***P < 0.0001

*Abbreviations*: *Cnr1*, Cannabinoid-1 receptor; *Cnr2*, Cannabinoid-2 receptor; *Dagla*, Diacylglycerol lipase alpha; *Daglb*, Diacylglycerol lipase beta; *Napepld*, *N*-acyl phosphatidylethanolamine phospholipase D. *Mgll*, Monoacylglycerol lipase; *Faah*, Fatty acid amide hydrolase; 2-AG, 2-arachidonoylglycerol; AA, arachidonic acid; AEA, anandamide or *N*-arachydonoylethanolamine; OEA, *N*-oleoylethanolamine; PEA, *N*-palmitoylethanolamine

**Fig. 3:**
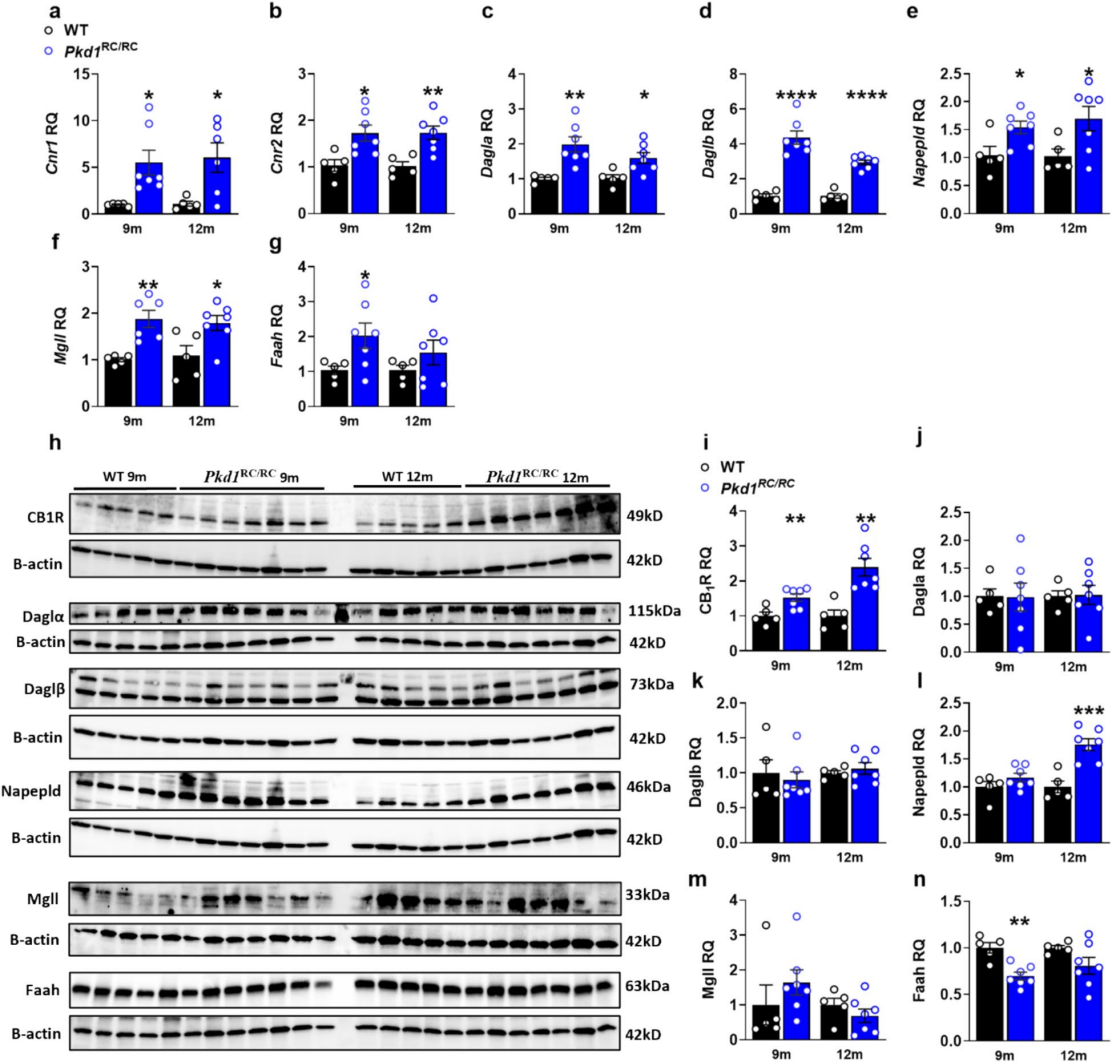
Temporal endocannabinoid system dysregulation during *Pkd1*^RC/RC^ disease progression. **a-g** Quantitative PCR analysis of ECS-related genes across disease stages (9 and 12 months) showing progressive upregulation of *Cnr1* **a** and *Cnr2* **b** transcripts, with corresponding changes in ECS metabolic enzymes: *Dagla* **c**, *Daglb* **d**, *Napepld* **e**, *Mgll* **f**, and *Faah* **g**. **h-n** Western blot analysis and quantification of ECS proteins across disease stages, demonstrating sustained CB_1_R protein elevation at 9 and 12 months **h, i**, stable 2-AG-related enzyme proteins (DAGLα, DAGLβ, MGLL; **h, j, k, m**), and stage-specific changes in AEA-related enzymes including elevated NAPEPLD at 12 months (**h, l**) and reduced FAAH at 9 months (**h, n**). Data represent mean ± SEM from WT (*n* = 5) and *Pkd1*^RC/RC^ (*n* = 6–7) per group per timepoint. Statistical analysis: unpaired *t*-test comparing *Pkd1*^RC/RC^ to age-matched WT. Statistical significance: *p < 0.05, **p < 0.01, ***p < 0.001, ****p < 0.0001 versus WT

Peak ECS dysregulation occurred at 9 months coinciding with active cyst expansion. During this stage, the majority of ECS-related enzymatic transcripts were upregulated (Table 1 and Fig. 3a-g). Although KW/BW ratio was already increased in *Pkd1*^RC/RC^ mice at early time points, the most pronounced and coherent ECS signal, characterized by *Cnr1*/CB_1_R upregulation together with selective AEA/OEA depletion and changes in AEA‑related enzymes, emerged at 9–12 months, coinciding with advanced cortical cystic remodeling and inflammatory/fibrotic activation. Notably, CB_1_R staining was increased in cystic regions, including cyst-lining epithelium and adjacent tubular structures, compared with WT kidneys, indicating broader cortical tubular upregulation rather than exclusive restriction to cyst-lining cells. This increase in CB_1_R protein abundance was elevated by 6 months and remained consistently increased thereafter (Supplementary Figure S4 and Fig. 3h-i), mirroring the transcriptional upregulation and preceding maximal ligand depletion. In contrast, 2-AG-related enzyme proteins (Dagla, Daglb, Mgll) remained largely unchanged throughout the study period (Fig. 3h, j-l), consistent with relatively stable 2-AG levels across disease stages (Table 1).

Transcriptional ECS changes were accompanied by reduced levels of *N*-ethanol-amines AEA and OEA in both sexes at 9 months; at 12 months, these reductions remain significant in females, whereas they are attenuated in males (Table 1). When directly comparing genotypes, AEA and OEA levels were similar between WT and *Pkd1*^RC/RC^ mice at 3 and 6 months, but became significantly lower in *Pkd1*^RC/RC^ mice at 9 months in both sexes, with these reductions persisting predominantly in females at 12 months, whereas 2‑AG levels remained largely comparable between WT and *Pkd1*^RC/RC^ at all time points (Table 1). Notably, AEA levels showed a trend toward reduction as early as 3 months in both sexes, preceding CB_1_R upregulation (which start occurring at 6 months), suggesting that ligand depletion may initiate ECS dysregulation. In both sexes, the largest number and magnitude of ECS transcript changes clustered at 9 months, whereas fewer transcripts remained significantly altered at 12 months, despite ongoing structural progression. This phasic behavior indicates that ECS transcriptional dysregulation peaks at an intermediate disease stage in both males and females, rather than increasing monotonically with KW/BW. By 12 months, male *Pkd1*^RC/RC^ mice had largely ‘caught up’ in terms of structural severity, while ECS transcripts showed a partial normalization in both sexes, in contrast to the persistent CB_1_R protein upregulation and reduction in AEA. These observations support a model in which ECS transcriptional changes represent a stage‑specific adaptive/maladaptive response, whereas protein and metabolite alterations persist into later stages.

Napepld protein expression was significantly increased at 12 months (Fig. 3h, m), while Faah displayed marked reduction at 9 months (Fig. 3h, n). Intriguingly, Faah protein decrease occurred despite divergent transcriptional changes (Table 1 and Fig. 3c), suggesting post-transcriptional regulation. Reinforcing this assumption, we found increased chromatin accessibility for all ECS-related genes in human PKD (Supplementary Figure S5).

Altogether, the temporal pattern in *Pkd1*^RC/RC^ mice demonstrates that ECS dysregulation progresses dynamically during ADPKD, with *Cnr1*/CB_1_R upregulation established by 6 months and selective *N*-acylethanolamines depletion emerging at 9 months. This temporal architecture closely parallels the late-stage ECS signature observed in human ADPKD kidneys, supporting the translational relevance of this mechanistic framework and indicating that ECS dysregulation is developmentally integrated into the disease process.

### ECS dysregulation correlates with renal function decline

**Fig. 4:**
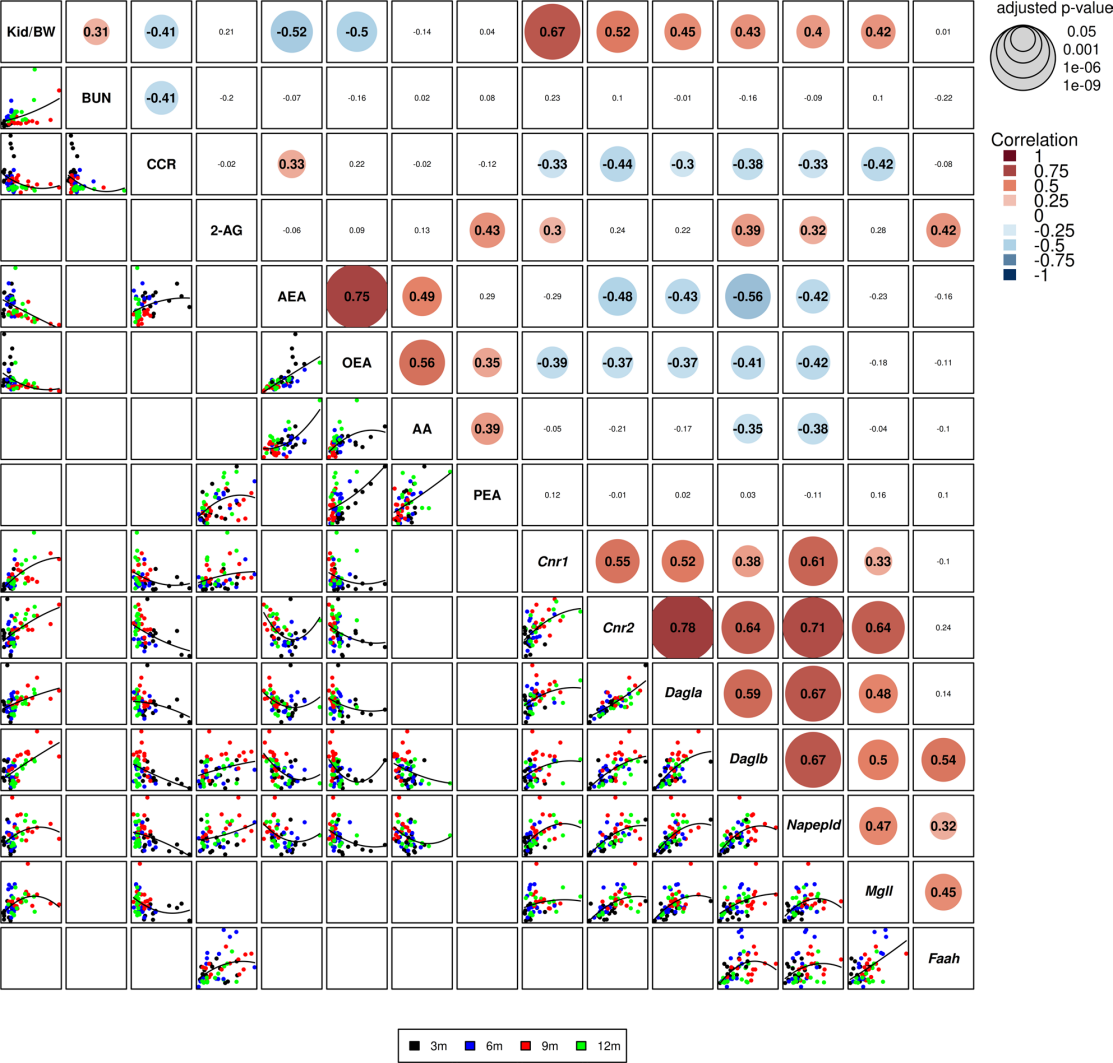
Endocannabinoid system dysregulation correlates kidney function decline. Correlation matrix (Spearman rank) between kidney function parameters (kidney-to-body weight ratio [KW/BW], blood urea nitrogen [BUN], creatinine clearance [CCr]), endocannabinoid ligands (2-arachidonoylglycerol [2-AG], anandamide [AEA], *N*-oleoylethanolamine [OEA], arachidonic acid [AA], *N*-palmitoylethanolamine [PEA]), and ECS receptor and enzyme transcriptional levels (*Cnr1, Cnr2, Dagla, Daglb, Napepld, Mgll, Faah*) across all disease stages (3, 6, 9, and 12 months (*n* = 7) per time point per sex) in mice only. Heatmap color intensity indicates correlation strength: red indicates positive correlation, blue indicates negative correlation. Circle sizes denote statistical significance when applicable: p < 0.05, p < 0.01, with Benjamini–Hochberg false discovery rate correction. Notable associations include a strong negative correlation between kidney AEA and KW/BW ratio, strong positive correlations between *Cnr1* expression and kidney enlargement/functional decline, and inverse associations between CCr and ECS gene expression, indicating coordinated dysregulation of ligands, receptors, and enzymes with disease severity, whereas 2-AG shows minimal correlation with these functional measures, consistent with its relatively stable levels over time

To investigate the relationship between ECS dysregulation and ADPKD severity, we performed Spearman rank correlation analyses linking ECS ligands, enzymes, and receptors to kidney function parameters across all *Pkd1*^RC/RC^ mice and time points (3, 6, 9, and 12 months). As expected for a progressive disease model, animals with the most advanced cyst burden (highest KW/BW, lowest CCr) exerted the greatest leverage on these relationships, while earlier‑stage animals clustered near intermediate values. Nevertheless, this analysis revealed robust and statistically significant associations between ECS activity and ADPKD progression (Fig. 4). KW/BW ratio exhibited a strong negative correlation with kidney AEA levels, driven predominantly by animals at 9–12 months that combined high KW/BW with marked AEA depletion, whereas many earlier‑stage *Pkd1*^RC/RC^ mice retained AEA levels overlapping the WT range. Among ECS transcripts, *Cnr1* showed the expected positive association with KW/BW and inverse association with CCr, while *Cnr2* displayed the steepest KW/BW correlation, consistent with its predominant expression in infiltrating immune cells and the progressive increase in inflammatory burden. Furthermore, CCr demonstrated inverse correlation with ECS receptor and enzyme expression, indicating that ECS dysregulation tracks closely with declining glomerular filtration. When correlations were re-examined after splitting the data by sex, sex-stratified analyses demonstrated broadly comparable correlation patterns in both males and females (Supplementary Figure S6); however, statistical significance appears more pronounced in females, reinforcing the robustness of these associations. Together, these findings support a model in which ECS ligand depletion, CB_1_R upregulation, and enzyme dysregulation are closely linked with ADPKD progression and may directly contribute to functional deterioration.

## Discussion

ADPKD is among the most prevalent genetic disorders, characterized by progressive cyst formation, leading to inflammation, fibrosis, and metabolic dysregulation. Although ADPKD pathogenesis has been extensively studied, the mechanisms integrating metabolic stress, epithelial injury, inflammation, and cyst expansion remain incompletely understood. The ECS has emerged as a critical regulator of renal metabolism, epithelial integrity, and inflammatory signaling (Nowak et al. 2018; Nowak et al. 2021; Kraus et al. 2016; Sas et al. 2015), yet its involvement in ADPKD has never been systematically examined. Prior work from our laboratory demonstrated that proximal tubular CB_1_R modulates AMPK (Udi et al. 2017) and mTORC1 (Hinden et al. 2022), two master metabolic regulators central to cyst growth and epithelial proliferation in ADPKD (Caplan 2022; Shillingford et al. 2006; Torres et al. 2017; Takiar et al. 2011). These observations led us to hypothesize that ECS dysfunction might contribute to ADPKD pathogenesis.

In this study, we provide the first comprehensive characterization of the ECS in ADPKD using an integrated approach spanning human multi-omics datasets, human kidney tissue biochemistry, and a longitudinal *Pkd1*^RC/RC^ mouse model. Across all platforms, we identify a coherent signature of ECS dysregulation marked by CB_1_R upregulation, impaired activity of key eCB-metabolizing enzymes, and pronounced depletion of eCB ligands AEA, OEA, and 2-AG. Together, these previously unrecognized findings reveal that ECS disruption plays a role in ADPKD pathophysiology.

A prominent and mechanistically significant finding is the dissociation between CB_1_R receptor expression and eCB ligand availability. Human transcriptomics and tissue analyses demonstrated robust *CNR1* upregulation and marked depletion of AEA, 2-AG, OEA, and AA. This pattern, elevated receptor with depleted ligands, is not simply "ECS activation" but rather represents a state of receptor sensitization in a ligand-depleted environment. Importantly, despite reduced *FAAH* expression (which degrades AEA), ADPKD kidneys exhibit profound AEA depletion. This paradox indicates that biosynthetic impairment or non-enzymatic consumption predominates over reduced degradation, suggesting upstream metabolic failure in eCB synthesis. Potential mechanisms include substrate (AA) depletion, impaired *NAPEPLD* enzymatic function, or diversion of lipid precursors toward alternative metabolic pathways during cyst-associated metabolic reprogramming.

The temporal data from *Pkd1*^RC/RC^ mice provide critical mechanistic insight: AEA levels trend downward as early as 3 months (though not statistically significant), preceding CB_1_R protein upregulation at 6 months. This temporal sequence suggests that ligand depletion may initiate compensatory receptor upregulation, establishing a feed-forward pathogenic loop. As endogenous CB_1_R agonists decline, the receptor may adopt constitutive activity or become hypersensitive to residual ligand, amplifying downstream signaling despite reduced absolute eCB levels. Alternatively, CB_1_R may interact with alternative endogenous ligands or undergo ligand-independent activation via mechanical stress, oxidative signals, or cross-talk with other G-protein-coupled receptors enriched in cystic epithelium.

The temporal patterns of ECS dysregulation also differ from a simple linear coupling to cyst burden. In both sexes, the strongest transcriptional signal in ECS genes was observed around 9 months, with partial attenuation at 12 months despite continued structural progression, while CB_1_R protein upregulation and AEA depletion persisted. Similar phasic transcriptional responses, with intermediate peaks and late declines, are well described in other chronic disease models (Loeser et al. 2013; Morsy et al. 2026). Thus, we interpret ECS transcriptional changes as reflecting a specific stage of cortical remodeling and injury, rather than a direct quantitative proxy of total cyst volume, with broadly similar temporal profiles in males and females but sex‑dependent differences in magnitude.

snRNA-seq revealed that ECS dysregulation is not uniformly distributed across the nephron but is enriched in proximal tubule-derived populations, particularly the FR-PTC state. FR-PTCs represent a maladaptive epithelial phenotype characterized by arrested differentiation, persistent injury signaling, metabolic stress, and proliferative capacity, biological features closely associated with regions of cyst remodeling (Kirita et al. 2020; Wu et al. 2019). However, based on the available snRNA‑seq datasets, these states cannot be assigned uniquely to cyst-lining epithelium versus injured non-cystic tubules. This cell-type specificity therefore suggests that ECS perturbation may actively contribute to maladaptive tubular epithelial dysfunction in regions associated with cyst formation, rather than being merely a passive consequence of kidney injury, but it does not by itself prove a cyst-epithelium-intrinsic program.

The enrichment of ECS dysregulation in FR-PTCs is mechanistically compelling given that CB_1_R signaling intersects with pathways central to this maladaptive state. CB_1_R activation suppresses AMPK (Udi et al. 2017), a negative regulator of mTORC1 and a promoter of cellular quiescence (Aguado et al. 2005; Molina-Holgado et al. 2002), while simultaneously potentiating mTORC1 activity (Hinden et al. 2022), which drives anabolic growth, protein synthesis, and proliferation (Song et al. 2020; Podrini et al. 2020; Caplan 2022; Schrier and Levi 2010; Ben-Sahra and Manning 2017). In the context of ADPKD, where elevated mTORC1 is causally linked to cyst expansion and is a clinical therapeutic target (Shillingford et al. 2006; Serra et al. 2010), CB_1_R-mediated metabolic reprogramming may directly fuel the FR-PTC phenotype and sustain cyst growth. Moreover, CB_1_R activation promotes pro-inflammatory and pro-fibrotic signaling (Tam et al. 2018; Udi et al. 2017; Hinden et al. 2022; Song et al. 2009; Han and Kim 2021), hallmarks of the FR-PTC transcriptional profile and drivers of progressive kidney dysfunction (Portilla et al. 2025).

To evaluate whether ECS dysregulation reflects ADPKD-specific pathology or represents a generic response to CKD, we analyzed snRNA-seq data from DKD patients. In contrast to ADPKD, DKD kidneys exhibited minimal transcriptional changes in *CNR1* or ECS-metabolizing enzymes, with no coordinated cell-type specific dysregulation. This disease comparison demonstrates that ECS disruption is not a universal feature of renal injury but appears tied to the unique metabolic and epithelial biology of ADPKD. This specificity strengthens the hypothesis that ECS dysregulation is mechanistically integrated into ADPKD progression rather than being an epiphenomenon of declining renal function. More broadly, ECS perturbations have been described in other forms of CKD, particularly in obesity‑ and diabetes‑related kidney disease, often in the form of elevated circulating eCBs and/or increased renal CB_1_R expression (Dao and François 2021; Udi et al. 2017; Arceri et al. 2023; Chua et al. 2019; Moradi et al. 2019). In this context, our data suggest that ADPKD is associated with a distinct ECS configuration, CB_1_R upregulation in the setting of AEA and *N*-acylethanolamine depletion, which is not recapitulated in DKD snRNA-seq analyzed with the same pipeline. Thus, ECS dysregulation should be viewed as an ADPKD-linked pattern within a wider spectrum of ECS changes in kidney diseases, rather than as a response unique to ADPKD in an absolute sense.

The *Pkd1*^RC/RC^ model is often characterized as predominantly distal/collecting-duct-driven at later stages, but lineage and morphologic studies indicate that cystogenesis in this model is stage-dependent and involves both proximal and distal tubular segments, with a substantial proximal tubule contribution in early disease and persistent cortical tubular dilation at later time points (e.g., 12 months) (Hopp et al. 2012). Longitudinal profiling in *Pkd1*^RC/RC^ mice revealed that ECS dysregulation develops progressively, with distinct temporal phases. Early disease (3 months) showed preserved ECS homeostasis with only subtle AEA trends. By 6 months, CB_1_R transcriptional and protein upregulation was firmly established, preceding maximal cyst burden. At 9–12 months, coinciding with accelerated cyst expansion and functional decline, *Pkd1*^RC/RC^ mice exhibited pronounced enzymatic dysregulation and significant AEA/OEA depletion. This staged progression indicates that ECS dysregulation is developmentally integrated into ADPKD pathogenesis, not merely a late-stage consequence.

Notably, the specific ECS components that change, and the direction and magnitude of transcript versus protein alterations, are not identical between human ADPKD kidneys and the *Pkd1*^RC/RC^ model. In mice, many ECS transcripts are increased at 9–12 months, whereas CB_1_R and selected AEA‑related enzymes show more selective protein changes; in human tissue, *CNR1* and some enzymatic transcripts are upregulated while *NAPEPLD* and *FAAH* are reduced and metabolic enzyme proteins are variably decreased. These differences likely reflect a combination of species‑specific regulation, post‑transcriptional and post‑translational control, and the fact that human samples represent terminal ESKD with extensive scarring and nephron loss, whereas the mouse data capture an earlier, pre‑terminal window. Therefore, our data not to imply perfect one‑to‑one concordance across species, but highlight a recurring, higher‑order pattern, CB_1_R upregulation with AEA/OEA depletion and AEA‑axis disruption, that appears in both systems when disease is advanced.

Sex-stratified analyses revealed that female *Pkd1*^RC/RC^ mice exhibit earlier and more pronounced ECS abnormalities than males, particularly with respect to *Cnr2* upregulation and sustained AEA/OEA depletion at 12 months. These findings highlight potential hormonal or metabolic modifiers of ECS regulation in ADPKD and suggest that sex may influence therapeutic responsiveness to ECS-targeted interventions, an important consideration for future preclinical and clinical studies.

Comprehensive correlation analyses demonstrated that ECS alterations, particularly AEA depletion and *Cnr1* upregulation, are most pronounced in animals with the largest kidneys and lowest CCr, indicating that the late‑stage ECS signature we describe is tightly linked to advanced structural and functional deterioration rather than linearly scaling with cyst burden at every time point. We also observed strong correlations between *Cnr2* and KW/BW, which likely reflect increasing immune cell infiltration and inflammatory activity, in line with the leukocyte‑restricted *CNR2* expression pattern seen in human snRNA‑seq data. Importantly, these associations were consistent in both male and female mice, reinforcing their biological robustness. While correlational, these data support a model in which ligand depletion, receptor upregulation, and enzyme dysregulation are functionally linked to ADPKD progression.

The consistent, cell-type-specific, and temporally dynamic ECS dysregulation we report here establishes peripheral CB_1_R signaling as a compelling therapeutic target in ADPKD. Peripherally restricted CB_1_R antagonists, compounds that block CB_1_R in kidney and other peripheral tissues without crossing the blood–brain barrier (Gammal et al. 2025; Tam et al. 2010; Tam et al. 2012; Hirsch et al. 2023), offer a strategy to interrupt pathogenic CB_1_R signaling while avoiding central nervous system side effects that limited first-generation CB_1_R antagonists. Preclinical studies from our laboratory and others have demonstrated that peripheral CB_1_R blockade ameliorates renal injury, fibrosis, and metabolic dysfunction in models of obesity-related kidney injury (Permyakova et al. 2023), DKD (Wilson et al. 2022), and TSC-induced cyst formation (Abergel et al. 2025). Given that CB_1_R activation drives mTORC1 (Hinden et al. 2022), suppresses AMPK (Udi et al. 2017), and promotes inflammation (Tam et al. 2018), all processes central to ADPKD cystogenesis (Caplan 2022; Margaria et al. 2020; Takiar et al. 2011), pharmacological CB_1_R antagonism may slow cyst growth, preserve tubular function, and delay disease progression. Importantly, CB_1_R inhibition would target the receptor itself, bypassing the complexities of ligand depletion and enzymatic dysregulation observed in our study. Genetic validation using tissue-specific CB_1_R deletion in *Pkd1*^RC/RC^ mice will be critical to establish causality and define the therapeutic window. Longitudinal intervention studies with peripherally restricted CB_1_R antagonists, initiated at early disease stages (e.g., 3 months in *Pkd1*^RC/RC^ mice), will determine whether modulating ECS activity can alter disease trajectory. Combination approaches targeting CB_1_R alongside established ADPKD therapies (e.g., vasopressin receptor antagonists (Hogan and Masyuk 2023; Torres et al. 2017; Torres et al. 2012)) may offer synergistic benefit by addressing orthogonal pathogenic pathways.

Several limitations of our study warrant consideration. First, human control kidney samples were predominantly medullary whereas ADPKD tissues were cortical, introducing potential regional bias. Second, the public single-nucleus RNA-seq datasets analyzed do not explicitly resolved a distinct “cyst-lining epithelium” cluster. Instead, epithelial cells are classified by nephron segment identity and injury-associated transcriptional states. As a result, we cannot unambiguously assign ECS dysregulation to cyst-lining cells versus surrounding injured tubular populations, and our FR-PTC findings should be interpreted as maladaptive tubular states enriched in ADPKD rather than as cyst-epithelium-specific per se. Nonetheless, the major ECS abnormalities were independently reproduced across human snRNA-seq, bulk microarray data, and our own ADPKD tissue RNA/protein/metabolite analyses, as well as in the longitudinal *Pkd1*^RC/RC^ mouse model, supporting that they reflect true disease-associated biology rather than sampling artifact. Third, human tissue analyses capture mainly late-stage ESKD, precluding assessment of early ECS dynamics; the *Pkd1*^RC/RC^ longitudinal model partially addresses this limitation by providing temporal resolution. Forth, although our data establish associations and a plausible temporal sequence between ECS alterations, cystic burden, and kidney dysfunction, they do not prove causality, and altered ECS activity could reflect a secondary response to fibrosis, inflammation, tubular injury or age‑related and segmental changes in nephron composition rather than a primary initiating event. Definitive testing will require genetic or pharmacological CB_1_R/ECS modulation in ADPKD models, which we identify as an important future direction. Finally, the *Pkd1*^RC/RC^ model harbors a hypomorphic rather than null mutation, which may influence disease kinetics and segmental cyst distribution (with a shift from predominantly proximal to more distal/collecting-duct involvement over time) (Hopp et al. 2012); however, this model recapitulates gradual-onset human ADPKD more faithfully than rapidly progressive orthologous models. The multi-layered human and experimental datasets presented here are intended as a resource and framework for such future mechanistic studies targeting CB_1_R/ECS in ADPKD.

In conclusion, this study identifies progressive ECS dysregulation, characterized by CB_1_R upregulation, enzymatic disruption, and eCB ligand depletion, as a novel and previously unrecognized factor in ADPKD pathophysiology. The enrichment of ECS dysregulation in FR-PTCs, the temporal dynamics paralleling disease progression, and the robust correlation with functional decline collectively support a model in which CB_1_R signaling may contribute to ADPKD progression. These findings establish peripheral CB_1_R antagonism as a mechanistically grounded and clinically viable therapeutic strategy warranting rigorous preclinical validation and potential translation to human ADPKD.

## Supplementary Information

Supplementary Material 1.

## Data Availability

The authors confirm that the data supporting the findings of this study are available in the article and its Supplementary Material.
