# Supplementary material for: Progressive endocannabinoid system dysregulation in autosomal dominant polycystic kidney disease
Source: Mol Med. 2026 Mar 16;32:64. doi: 10.1186/s10020-026-01457-w (PMC13104467; doi:10.1186/s10020-026-01457-w)
Supplement: Supplementary file 1 — Supplementary Material 1. [file 10020_2026_1457_MOESM1_ESM.pdf]

Supplementary Information

Progressive Endocannabinoid System Dysregulation in Autosomal Dominant Polycystic Kidney Disease

Shridhar Betkar[1], Alina Nemirovski[1], Shmuel Ruppo[2], Liad Hinden[1,*], and Joseph Tam[1,*]

[1]Obesity and Metabolism Laboratory, Institute for Drug Research, School of Pharmacy, Faculty of Medicine, The Hebrew University of Jerusalem, Jerusalem, Israel; [2]Info-CORE, Bioinformatics Unit of the I-CORE, The Hebrew University of Jerusalem, Jerusalem, Israel.

Supplementary Table 1. MRM transitions for eCBs measurements in ESI+

Analytes	Molecular ion $[M+H]^+$ [m/z]	Fragment [m/z]	DP [volts]	CE [volts]	CXP [volts]
2-AG	379.2	287.1 (quantifier)	70	19	14
		91 (qualifier)	70	67	10
AEA	348.2	287.1 (quantifier)	26	13	16
		62 (qualifier)	26	13	8
PEA	300.3	283.2 (quantifier)	130	19	24
		62 (qualifier)	130	17	8
AA	305.3	91 (quantifier)	1	49	10
		287.1 (qualifier)	1	13	22
OEA	326.3	61.9 (quantifier)	146	21	24
		309.1 (qualifier)	146	21	42
d_4-AEA	352.3	287.1 (quantifier)	66	15	20
		66 (qualifier)	66	21	8
d_5-AG	384.3	287.1 (quantifier)	26	19	14
		91 (qualifier)	26	67	10

2-AG, 2-arachidonoylglycerol; AEA, anandamide or N-arachidonoylethanolamine; OEA, *N*-oleoylethanolamine; PEA, *N*-palmitoylethanolamine; AA, arachidonic acid. DP- declustering potential; CE- collision energy; CXP- Collision Cell Exit Potential

Supplementary Table 2. Human primers

Gene	Forward primer (5'-3')	Reverse primer (5'-3')
CNR1	AAGCCCGCATGGACATTAGGTTAG	AGCAGAGGGCCCCAGCAGAT
DAGLA	CCATCTTCCTCTTTCTCCT	CTCGTGCGGGTTATAGAC
DAGLB	TCAGGTGCTACGCCTTCTC	TCACACTGAGCCTGGGAATC
FAAH	CACACGCTGGTTCCCTTCTT	GGGTCCACGAAATCACCTTTGA
MGLL	GGAAACAGGACCTGAAGACC	ACTGTCCGTCTGCATTGAC
NAPEPLD	ACTGGTTATTGCCCTGCTTT	AATCCTTACAGCTTCTTCTGGG
HPRT1	CATTATGCTGAGGATTTGGAAAGG	CTTGAGCACACAGAGGGCTACA

#

Supplementary Table 3. Mouse primers

Gene	Forward primer (5'-3')	Reverse primer (5'-3')
Cnr1	CCGCAAAGATAGTCCCAATG	AACCCCACCCAGTTTGAAC
Cnr2	CTGCAGCTCTTGGGACCTAC	TGTCCCAGAAGACTGGGTGT
Col1	TTCTCCTGGCAAAGACGGACTCAA	GGAAGCTGAAGTCATAACCGCCA
Col3	ACAGCAAATTCACTTACACAGTTC	CTCATTGCCTTGCGTGTTT
Dagla	GTCCTGCCAGCTATCTTCCTC	CGTGTGGGTTATAGACCAAGC
Daglb	AGCGACGACTTGGTGTTCC	GCTGAGCAAGACTCCACCG
Faah	GTATCGCCAGTCCGTCATTG	GCCTATACCCTTTTTCATGCCC
Fn1	ATGTGGACCCCTCCTGATAGT	GCCCAGTGATTTCAGCAAAGG
Il6	GACAACCACGGCCTTCCCTA	GCCTCCGACTTGTGAAGTGGT
Il18	GACTCTTGCGTCAACTTCAAGG	CAGGCTGTCTTTTGTCAACGA
Ip10	GGATGGCTGTCCTAGCTCTG	TGAGCTAGGGAGGACAAGGA
Lcn2	TTTCACCCGCTTTGCCAAGT	GTCTCTGCGCATCCCAGTCA
Mcp1	GCATTAGCTTCAGATTTA	TTAAAAACCTGGATCGGAACCAA
Mgll	ACCATGCTGTGATGCTCTCTG	CAAACGCCTCGGGGATAACC
Napepld	ACGTCCTCCTCTAGTCTGTAATC	AGCGCCAAGCTATCAGTATCC
Tgfb	GCGGACTACTATGCTAAAGAGG	GTAGAGTTCCACATGTTGCTCC
Tnfa	QT00104006 QuantiTect Primer Assays (Qiagen, Germany)	
b-Actin	GGCTGTATTCCCCTCCATCG	CCAGTTGGTAACAATGCCATGT
Ubc	GCCCAGTGTTACCACCAAGA	CCCATCACACCCAAGAACA

Supplementary Table 4. List of antibodies used for Western blotting

Target	Antibody (Product code, Source)	Dilution
**CB1R**	0577421-1, Cayman	1:1000
**DAGLA**	ab81984, Abcam	1:500
**DAGLB**	ab191159, Abcam	1:500
**FAAH**	ab54615, Abcam	1:5000
**MAGL**	ab228598, Abcam	1:5000
**NAPEPLD**	ab95397, Abcam	1:200
**β-Actin**	ab49900, Abcam	1:30,000
**Anti-rabbit**	ab97085, Abcam	1:5000
**Anti-mouse**	ab98799, Abcam	1:2500
**Anti-goat**	ab97110, Abcam	1:5000

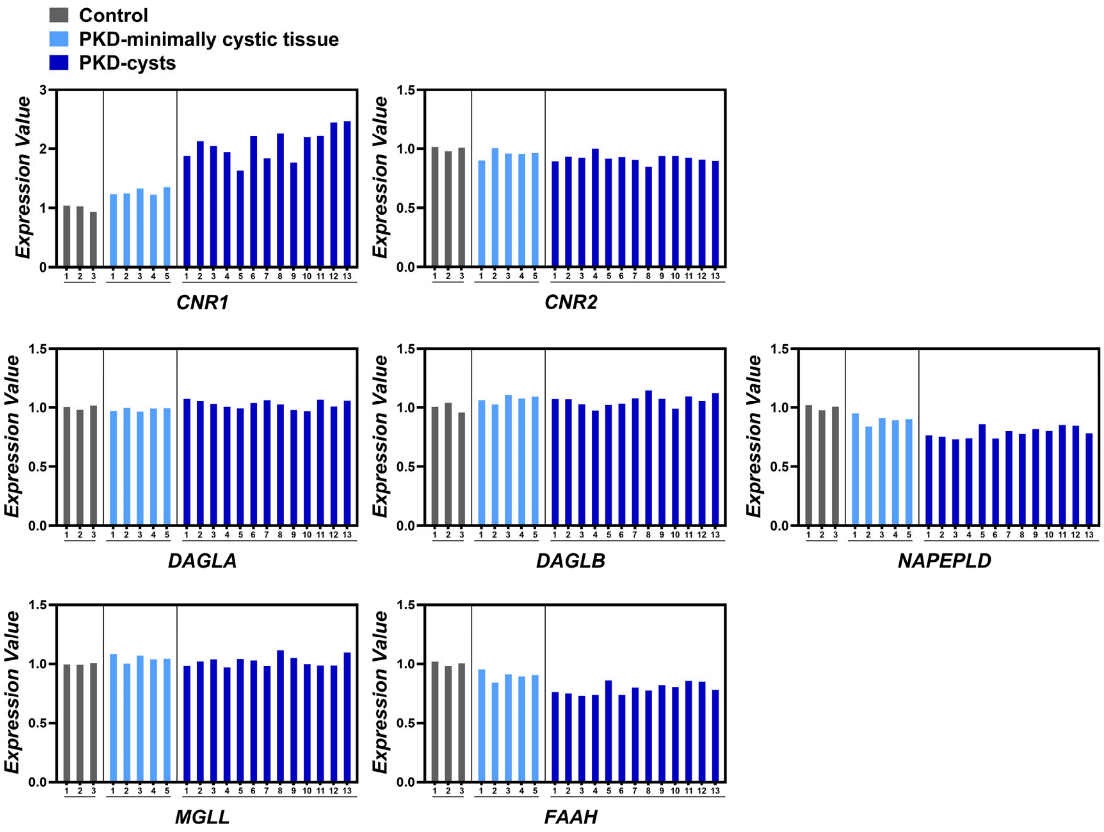

Supplementary Figure S1. Expression patterns of ECS genes across disease severity in human ADPKD. Individual sample-level expression values for seven ECS-related genes (*CNR1, CNR2, DAGLA, DAGLB, NAPEPLD, MGLL, FAAH*) from the GSE7869 microarray dataset. Human kidney tissue samples (n = 19 total) were categorized into three disease severity groups: healthy control cortex (n = 6), minimally cystic ADPKD tissue (PKDm, n = 5), and advanced cystic ADPKD tissue (PKD, n = 8). Each bar represents the normalized expression value of an individual sample. Data demonstrate consistent stepwise increases in *CNR1* expression and progressive reductions in AEA-metabolizing enzymes *NAPEPLD*, *MGLL*, and *FAAH* across disease severity stages, supporting the progressive ECS dysregulation observed in main **Figure 1a**. Expression values were normalized against healthy control samples and analyzed using GraphPad Prism (v10.4) with statistical comparisons by Benjamini-Hochberg false discovery rate-corrected analysis via GEO2R platform.

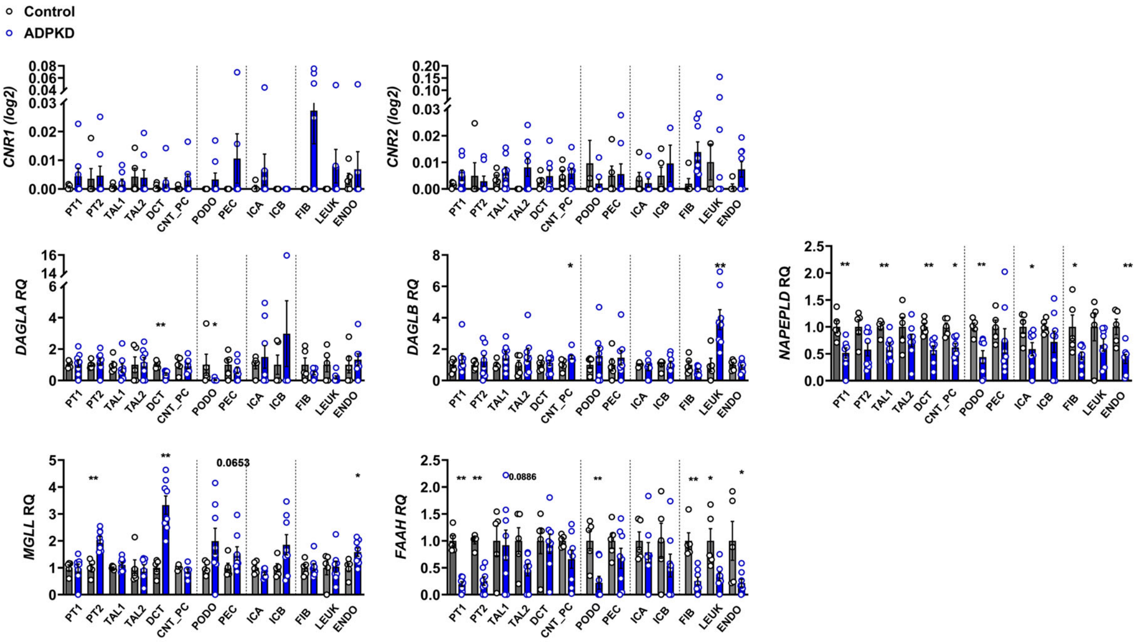
Control
ADPKD
CNR1 (log2)
CNR2 (log2)
DAGLA RQ
DAGLB RQ
NAPEPLD RQ
MGLL RQ
FAAH RQ
PT1 PT2 TAL1 TAL2 DCT CNT_PC PODO PEC ICA ICB FIB LEUK ENDO
0.0653
0.0886

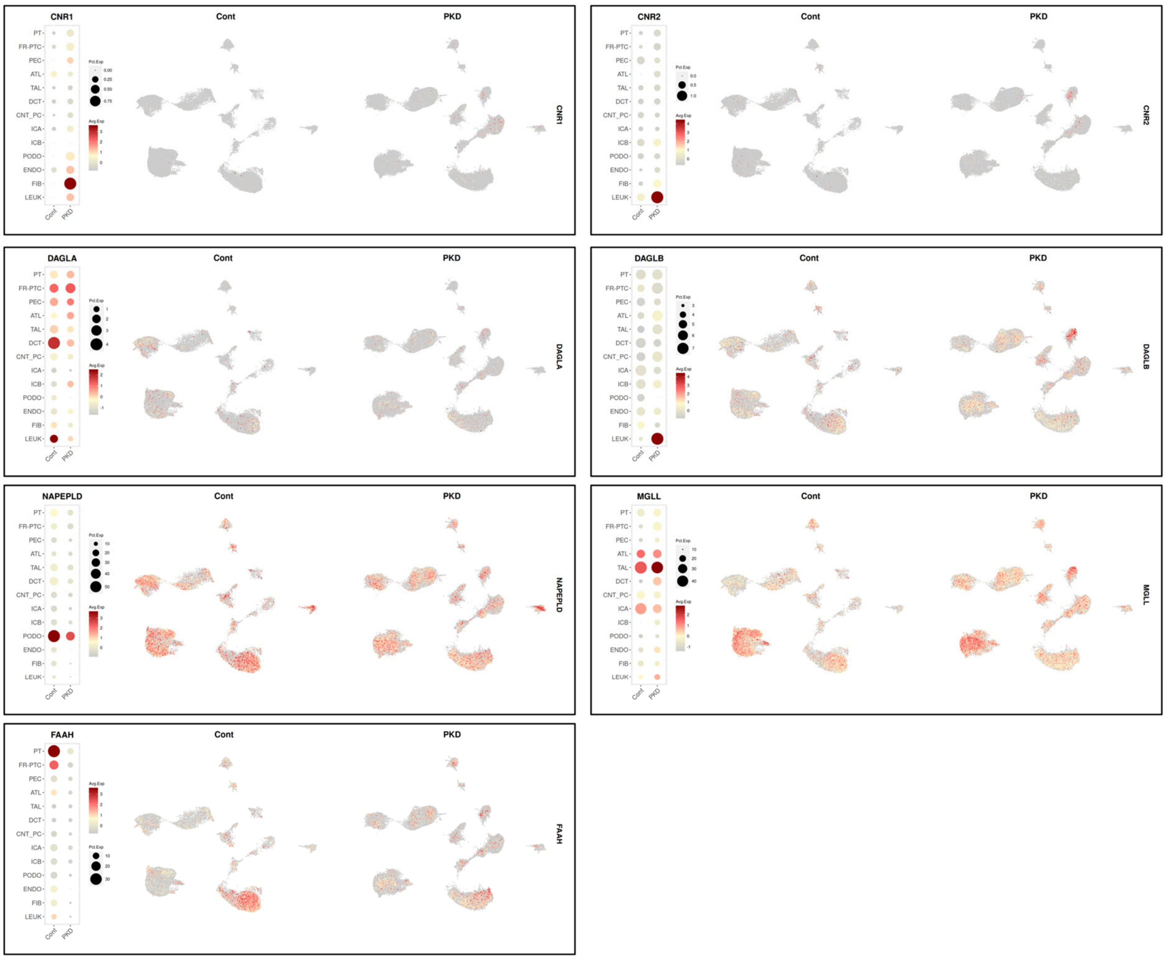
CNR1
CNR2
DAGLA
DAGLB
NAPEPLD
MGLL
FAAH
Cont
PKD

Supplementary Figure S2. Cell-type-resolved ECS gene expression in human ADPKD kidney. (Top panel) Pseudo-bulk single-nucleus RNA-sequencing (snRNA-seq) analysis of ECS gene expression (*CNR1, CNR2, DAGLA, DAGLB, NAPEPLD, MGLL, FAAH*) across annotated kidney cell populations from ADPKD patients (n = 8, blue) and healthy controls (n = 5, grey). Data represent log_2-normalized average expression values per sample within each annotated cell type. (Bottom panel) UMAP feature plots and complementary dot plots showing spatial distribution and relative expression of each ECS gene across the full repertoire of kidney cell types. Dot size indicates the percentage of cells expressing each gene within a cluster, and color intensity reflects average transcript abundance. Cell cluster annotation was performed using established kidney marker genes and cross-referenced with the Humphreys Lab Kidney Interactive Transcriptomics (KIT) portal. Identified populations include: proximal tubule (PT), failed-repair PT (FR-PTC), parietal epithelial (PEC), thick ascending limb (TAL), distal convoluted tubule (DCT), principal cells (PC), intercalated cells (ICA, ICB), podocytes (PODO), endothelial (ENDO), fibroblast (FIB), and leukocyte (LEUK) clusters.

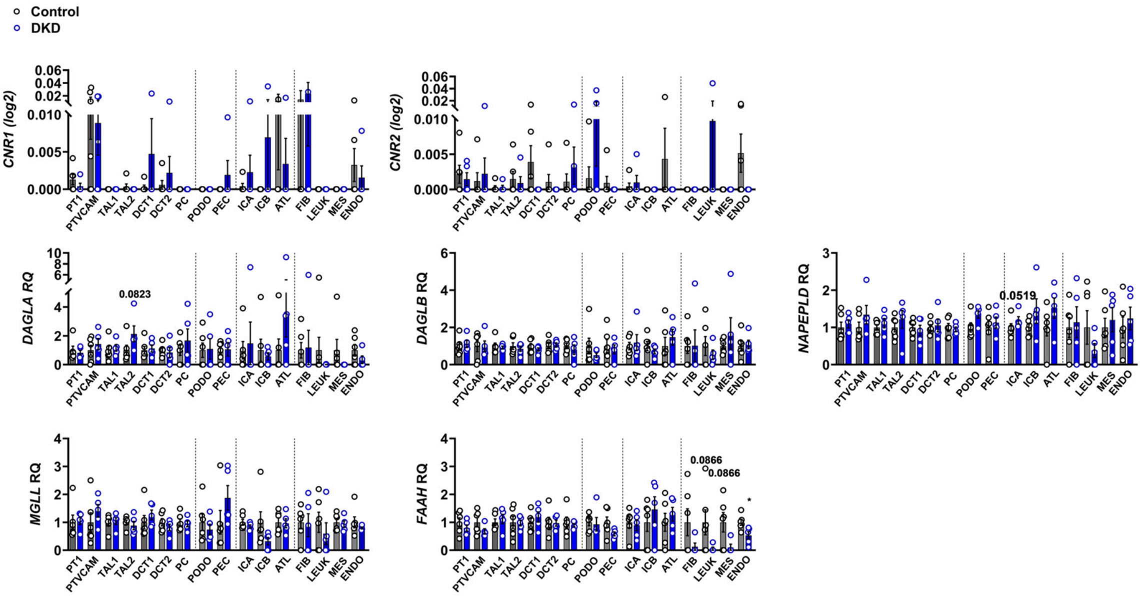
Control
DKD
CNR1 (log2)
CNR2 (log2)
DAGLA RQ
DAGLB RQ
NAPEPLD RQ
MGLL RQ
FAAH RQ
0.0823
0.0519
0.0866
0.0866
PT1
PTVCAM
TAL1
TAL2
DCT1
DCT2
PC
PODO
PEC
ICA
ICB
ATL
FIB
LEUK
MES
ENDO

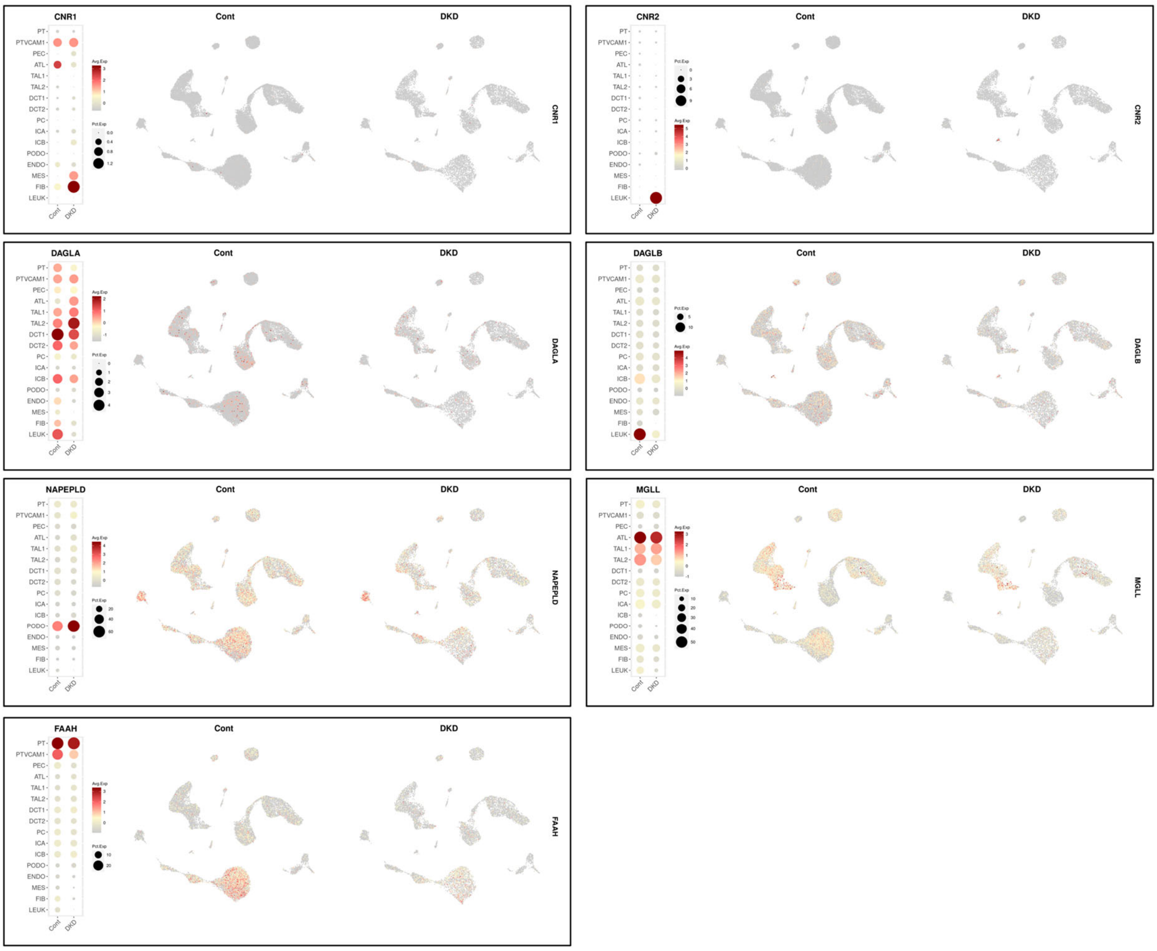
CNR1
CNR2
DAGLA
DAGLB
NAPEPLD
MGLL
FAAH
Cont
DKD

Supplementary Figure S3. ECS gene expression is preserved in diabetic kidney disease. (Top panel) Pseudo-bulk snRNA-seq analysis of the same seven ECS genes (*CNR1, CNR2, DAGLA, DAGLB, NAPEPLD, MGLL, FAAH*) across annotated kidney cell populations from diabetic kidney disease (DKD) patients (n = 5, blue) and healthy controls (n = 6, grey). Data represent log_2-normalized average expression per cell type per sample. (Bottom panel) UMAP feature plots and dot plots illustrate spatial distribution and transcript abundance of each ECS gene across all DKD kidney cell clusters. Dot size reflects the proportion of expressing cells, and color intensity represents average transcript levels. Cell cluster annotation was performed using established kidney marker genes and the Humphreys Lab KIT portal. Identified populations include: proximal tubule (PT), VCAM1^{+} PT (PTVCAM1), parietal epithelial (PEC), thick ascending limb (TAL1, TAL2), distal convoluted tubule (DCT1, DCT2), principal cells (PC), intercalated cells (ICA, ICB), podocytes (PODO), endothelial (ENDO), fibroblast (FIB), mesangial (MES), and leukocyte (LEUK) clusters.

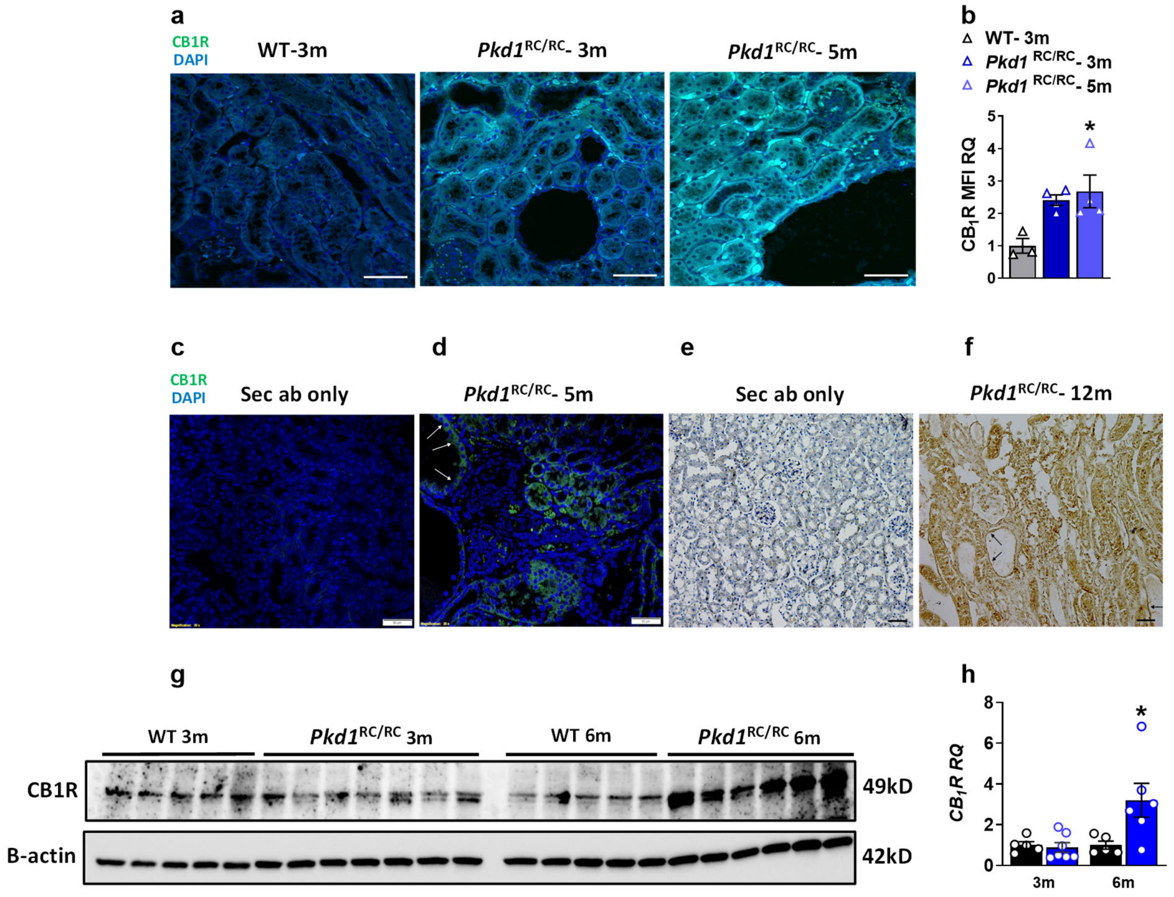

Supplementary Figure S4. CB_1R protein elevation in *Pkd1*$^{RC/RC}$ mice. (**a, b**) Representative frozen tissue immunofluorescence images and corresponding quantification of kidney tissue from WT control and *Pkd1*$^{RC/RC}$ mice showing CB_1R staining (ImmunoGenes Ltd. CB_1R antibody, green) and DAPI nuclear counterstain (blue). Enhanced CB_1R signal intensity is observed in cyst-lining epithelial and tubular regions of *Pkd1*$^{RC/RC}$ kidneys compared with normal tubular epithelium in WT controls. (**c**) Control immunofluorescence image with secondary antibody only. (**d**) Representative immunofluorescence image with white arrows indicating cyst-lining epithelial cells exhibiting CB_1R expression. Scale bars: 50 µm. (**e, f**) Paraffin embedded immunohistochemistry control and CB_1R staining (Cayman CB_1R antibody) in 12-month-old *Pkd1*$^{RC/RC}$ kidney sections demonstrating CB_1R expression in cyst-lining epithelium (arrows) as well as broader tubular epithelial compartments. Scale bars: 50 µm. (**g**) Representative Western blot analysis showing CB_1R protein expression in whole kidney lysates from WT and *Pkd1*$^{RC/RC}$ mice at 3 and 6 months. β-actin was used as a loading control. (**h**) Quantitative densitometry of CB_1R protein bands normalized to β-actin demonstrates significant elevation of CB_1R protein beginning at 6 months in *Pkd1*$^{RC/RC}$ mice. Data are presented as mean ± SEM; individual points represent biological replicates (WT n = 3-5 and *Pkd1*$^{RC/RC}$ n = 5-7 per group). Statistical analysis: unpaired *t*-test; $*p < 0.05$ versus age-matched WT controls.

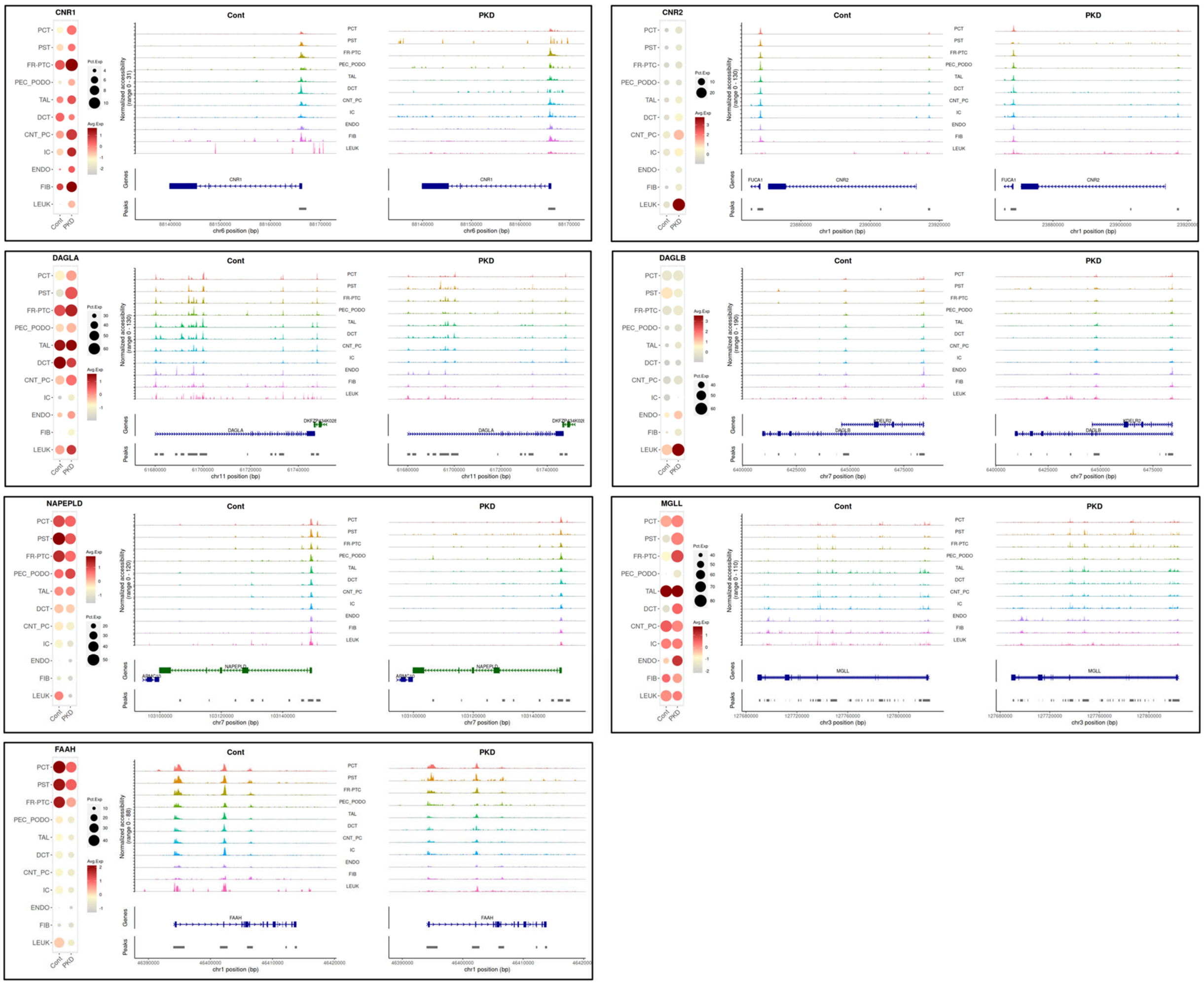

Supplementary Figure S5. Chromatin accessibility profiles of key ECS-related genes in human control and ADPKD kidneys. Genome browser tracks display aggregated single-nucleus ATAC-seq signal across major renal cell populations from ADPKD patients (n = 8) and healthy controls (n = 5) for *CNR1, CNR2, DAGLA, DAGLB NAPEPLD, MGLL, FAAH*, and loci. Increased accessibility across promoter and gene body regions is observed for multiple ECS genes in ADPKD, particularly within proximal tubule and epithelial lineages, indicating a transcriptionally permissive chromatin state.

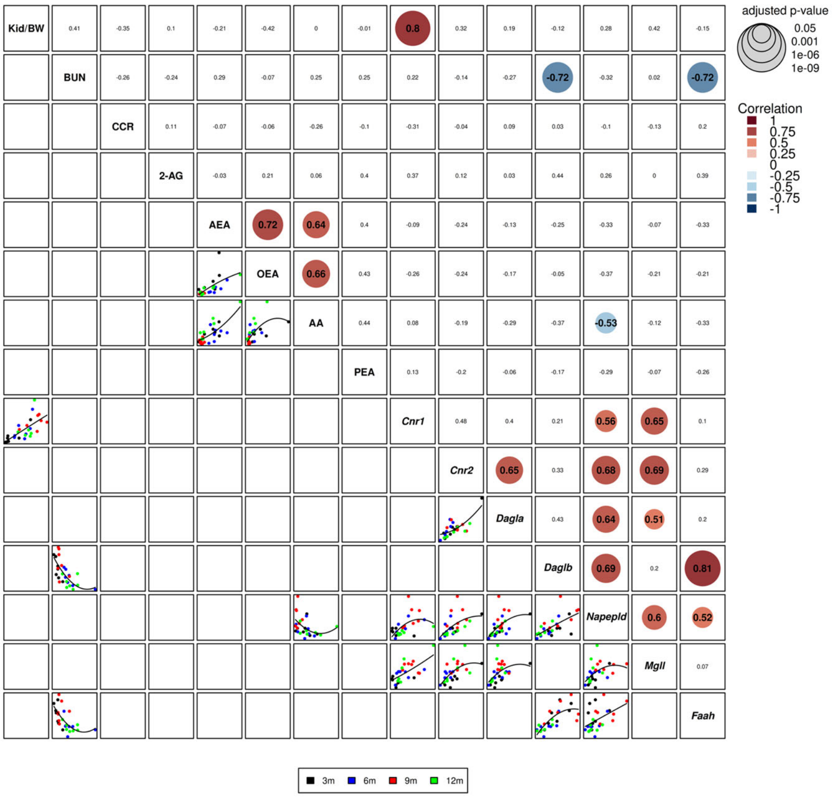
Kid/BW
BUN
CCR
2-AG
AEA
OEA
AA
PEA
Cnr1
Cnr2
Dagla
Daglb
Napepld
Mgll
Faah
adjusted p-value
0.05
0.001
1e-06
1e-09
Correlation
1
0.75
0.5
0.25
0
-0.25
-0.5
-0.75
-1
3m
6m
9m
12m

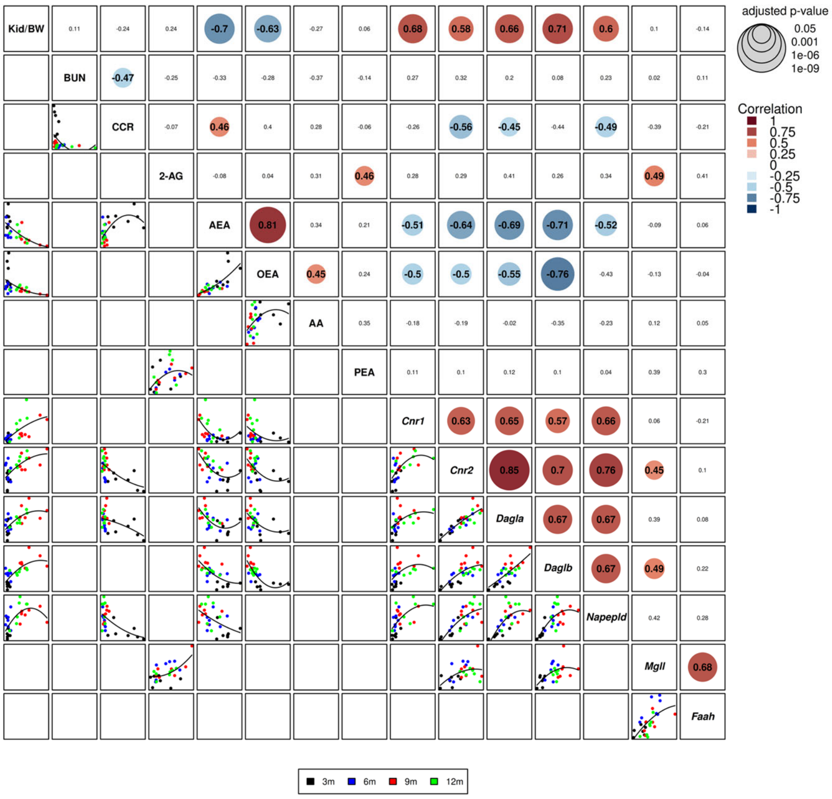
Kid/BW
BUN
CCR
2-AG
AEA
OEA
AA
PEA
Cnr1
Cnr2
Dagla
Daglb
Napepld
Mgll
Faah
adjusted p-value
0.05
0.001
1e-06
1e-09
Correlation
1
0.75
0.5
0.25
0
-0.25
-0.5
-0.75
-1
3m
6m
9m
12m

Supplementary Figure S6. Sex-stratified correlations between ECS components and kidney function. Spearman rank correlation matrices comparing endocannabinoid system components with kidney function parameters stratified by sex in *Pkd1*$^{RC/RC}$ mice across all disease stages (3, 6, 9, and 12 months (n = 7) per time point per sex). Male mice (**Upper panel**), Female mice (**Lower panel**): Correlation matrix showing associations between kidney function measures [kidney-to-body weight ratio (KW/BW), blood urea nitrogen (BUN), creatinine clearance (CCr)] and endocannabinoid ligands (2-arachidonoylglycerol [2-AG], anandamide [AEA], *N*-oleoylethanolamine [OEA], arachidonic acid [AA], *N*-palmitoylethanolamine [PEA]), as well as ECS-metabolizing enzymes (*Dagla, Daglb, Napepld, Mgll, Faah*) and receptors (*Cnr1, Cnr2)*.
